# Drug-Resistant Gram-Positive Cocci as Etiological Factors of Cardiac Implantable Electronic Device Infections—Data from the EXTRACT Registry

**DOI:** 10.3390/antibiotics15040345

**Published:** 2026-03-27

**Authors:** Danuta Łoboda, Sylwia Gładysz-Wańha, Michał Joniec, Eugeniusz Piłat, Robert D. Wojtyczka, Beata Sarecka-Hujar, Julia Staroń, Denis Swolana, Michał Gibiński, Karolina Simionescu, Sławomir Wilczyński, Krzysztof S. Gołba

**Affiliations:** 1 Department of Electrocardiology and Heart Failure, Medical University of Silesia, 40-635 Katowice, Poland; mgibinski@sum.edu.pl (M.G.); ksimionescu@sum.edu.pl (K.S.); kgolba@sum.edu.pl (K.S.G.); 2 Department of Electrocardiology, Upper-Silesian Medical Centre in Katowice, 40-635 Katowice, Poland; 3 Doctoral School, Medical University of Silesia, 40-055 Katowice, Poland; 4 Department of Microbiology, Faculty of Pharmaceutical Sciences in Sosnowiec, Medical University of Silesia, 41-200 Sosnowiec, Poland; rwojtyczka@sum.edu.pl (R.D.W.); dswolana@sum.edu.pl (D.S.); 5 Department of Basic Biomedical Science, Faculty of Pharmaceutical Sciences in Sosnowiec, Medical University of Silesia, 41-200 Sosnowiec, Poland; bsarecka-hujar@sum.edu.pl (B.S.-H.); swilczynski@sum.edu.pl (S.W.)

**Keywords:** cardiac implantable electronic device, antimicrobial resistance, Gram-positive cocci, infective endocarditis, multidrug resistance, pocket infection, pocket erosion, transvenous lead extraction

## Abstract

Introduction: Bacterial multidrug resistance (MDR) drives treatment with expensive, toxic, or pharmacokinetically suboptimal antibiotics. Objectives: To assess the prevalence of MDR Gram-positive cocci among isolates from cardiac implantable electronic device (CIED) infections at a Polish reference center. Methods: Data come from the “EXTRACT” registry (ClinicalTrials.gov ID NCT05775783), which covers 702 transvenous lead extraction procedures. Blood samples and intraoperative swabs were collected from participants with CIED infection. Results: From 209 cases with isolated pocket infection (PI) (107, 51.2%) or systemic infections (102, 48.8%), 263 Gram-positive cocci were cultured. They were: coagulase-negative staphylococci (CoNS) (177, 67.3%), *Staphylococcus aureus* (62, 23.6%), enterococci (15, 5.7%), streptococci (8, 3.0%), and others (1, 0.4%). The highest MDR rate was among CoNS (46.9%). CoNS exhibited methicillin resistance (MR-CoNS) in 55.9% with co-resistance to macrolides (73.2%), lincosamides (51.0%), fluoroquinolones (56.1%), aminoglycosides (41.4%), tetracyclines (29.6%), and co-trimoxazole (29.3%). Resistance to daptomycin (5.3%) and linezolid (2.0%) in MR-CoNS was rare. The frequency of MDR *S. aureus* was 8.1%. Methicillin resistance in *S. aureus* (MRSA, 6.5%) co-occurred with resistance to macrolides/lincosamides and fluoroquinolones (100% for both) or linezolid (25.0%). All MDR staphylococci were vancomycin-susceptible. High-level aminoglycoside resistance (HLAR) in *Enterococcus faecalis* (53.8%) was accompanied by levofloxacin co-resistance (66.7%). Conversely, *E. faecium* HLAR (50.0%) strains showed 100.0% β-lactam resistance. Vancomycin-resistant enterococci (VRE) accounted for 6.7%; the VRE *E. faecium* strain was tigecycline- and linezolid-susceptible. Among viridans group streptococci, β-lactam and lincosamides resistance was common (40.0% for both), with 50.0% of co-resistance. Conclusions: Epidemiological data may improve the effectiveness of empirical antibiotic therapy for CIED-related infections.

## 1. Introduction

In Poland, approximately 44,000 cardiac implantable electronic devices (CIEDs) were implanted in 2024, with the highest number in the Masovian and Silesian voivodeships [1]. Increased life expectancy is driving the number of implantations. The lifetime risk of concomitant infection ranges from 1.19% to 3.35% [2], depending on device type, procedural complexity, prior CIED-related infection, and patient comorbidities [2,3,4,5].

Local CIED infection usually results from surgical wound contamination during device implantation or replacement (early infections) or from pressure exerted by the generator or superficial lead segments on the skin, leading to pocket erosions and secondary bacterial colonization (late infections). Early infections are associated with poor antibiotic prophylaxis, prolonged procedures, early reoperation, and pocket hematoma. Late pocket infections are mainly due to shallow generator placement and little subcutaneous tissue, leading to tissue ischemia [6,7]. Infection can then spread along lead segments to the tricuspid valve and right heart chambers, causing CIED-related infective endocarditis (CIED-IE). Another route is bloodstream infection (bacteremia) from a distant site, such as a contaminated vascular catheter, surgical site, or infection in the respiratory, urinary, or abdominal tract. Bacteria often settle on damaged lead insulation, which promotes adhesion and biofilm formation. Lead abrasions mainly arise from intracardiac friction between leads or with intracardiac structures, including the superior vena cava and tricuspid valve. This can contribute to CIED-IE and formation of vegetation even without pocket infection [5]. Patient comorbidities also strongly influence CIED infection risk [6,7].

The etiological agents of CIED infections are predominantly Gram-positive cocci [8,9]. In Poland, *Staphylococcus aureus* accounts for 20–30% of CIED-related infections. Coagulase-negative staphylococci (CoNS), as opportunists, are also frequently encountered in healthcare-associated infections, due to their ability to colonize inert materials such as prosthetic valves, CIEDs, and venous catheters. They are cultured from 25 to 35% of CIED and 20–44% of bloodstream infections. Enterococci, viridans group streptococci (VGS), and *Streptococcus pneumoniae* are the etiological agents of another 15% CIED infections. Gram-positive (mainly *Cutibacterium acnes* and *Corynebacterium* spp.) and Gram-negative bacilli comprise the remaining, less common positive cultures [3,5,9,10].

The rise in antimicrobial resistance (AMR) among Gram-positive cocci is concerning [11,12,13,14,15,16]. AMR can develop through various mechanisms, including the induction by environmental antimicrobial agents, spontaneous mutations in chromosomal genes, and the acquisition of resistance through horizontal gene transfer, including interspecies transfer [17]. During such transfers, mobile genetic elements—plasmids, transposons, integrons, or chromosomal gene cassettes—carrying multidrug-resistance (MDR) genes are shared. Some Gram-positive cocci, i.e., methicillin- and vancomycin-resistant *S. aureus* (MRSA, VRSA), vancomycin-resistant enterococci (VRE), and penicillin-non-susceptible *S. pneumoniae* are monitored by the European Antimicrobial Resistance Surveillance Network (EARS-Net) in all European Union/European Economic Area countries [18].

The study aims to assess the prevalence of MDR Gram-positive cocci among strains isolated during transvenous lead extraction (TLE) procedures performed on patients with CIED-related infections at a large reference center in the Silesian Voivodeship, Poland.

## 2. Results

### 2.1. Cohort Characteristics

A total of 209 patients with a mean age of 66.24 (SD 12.99), 56 (26.8%) women, with evidence of CIED infection eligible for TLE procedure, constituted the study group. The reasons for TLE procedure were isolated pocket infection/pocket erosion (PI) in 107 cases (51.2%), PI with bacteremia/CIED-IE in 55 cases (26.3%), and isolated bacteremia/CIED-IE in 47 cases (22.5%).

Table 1 presents baseline characteristics of the study group by the infection type.

### 2.2. Characteristics of Gram-Positive Cocci Isolates

From 209 patients, 263 Gram-positive cocci were cultured, including CoNS (177 strains, 67.3%), *S. aureus* (62 strains, 23.6%), *Enterococcus* spp. (15 strains, 5.7%), group B (GBS), group C, and VGS (8 strains, 3.0%), and *Aerococcus viridans* (1 strain, 0.4%). *S. aureus* (in 82.3%) and CoNS (in 88.7%) were isolated mainly from infections originating in the generator pocket, whereas 40% of streptococci and enterococci were from CIED-IE without PI.

Table 2 shows isolated species by the infection type.

### 2.3. AMR Patterns Throughout the Observation Period

Table 3, Table 4 and Table 5 present the resistance patterns of all cultured bacterial isolates, grouped by species. The prevalence of β-lactam-resistant strains remained at a stable trend throughout the observation period (2016–2025); *p* = 0.78 for MRSA, *p* = 0.58 for methicillin-resistant CoNS (MR-CoNS), *p* = 0.19 for ampicillin-resistant enterococci, and *p* = 0.47 for penicillin-resistant streptococci. A consistent resistance rate to macrolides, lincosamides, and fluoroquinolones was observed in both *S. aureus* (*p* = 0.48, *p* = 0.69, and *p* = 0.99, respectively) and CoNS (*p* = 0.97, *p* = 0.21, and *p* = 0.21, respectively). Similarly, the percentage of high-level aminoglycoside resistance (HLAR) in enterococci did not show an increasing trend (*p* = 0.87). Supplementary Figures S1–S4 show rates of AMR strains during the observation period.

### 2.4. MDR Strains

The following resistance phenotypes were identified in Gram-positive cocci [16,17,19,20,21]; Table 6:

The highest MDR rate was observed among CoNS (46.9%). CoNS exhibited methicillin resistance in 55.9% of cases. MR-CoNS were co-resistant to macrolides (73.2%), lincosamides (51.0%), fluoroquinolones (56.1%), aminoglycosides (41.4%), tetracyclines (29.6%), and co-trimoxazole (29.3%) but rarely to daptomycin (5.3%), and linezolid (2.0%). All MR-CoNS were vancomycin-susceptible. Among *S. aureus*, methicillin resistance was observed in only 6.5%. It co-occurred with resistance to macrolides/lincosamides and fluoroquinolones (100% for both), or linezolid (25.0%). However, the overall frequency of MDR strains was relatively low at 8.1%. All MRSA were susceptible to rifampicin, vancomycin, and daptomycin, as well as gentamycin, tetracycline, and tigecycline. MRSA and MR-CoNS isolates showed a higher MDR rate than methicillin-susceptible *S. aureus* (MSSA) and CoNS (MS-CoNS) (*p* < 0.001 for both); Supplementary Table S2.

**Figure 1:**
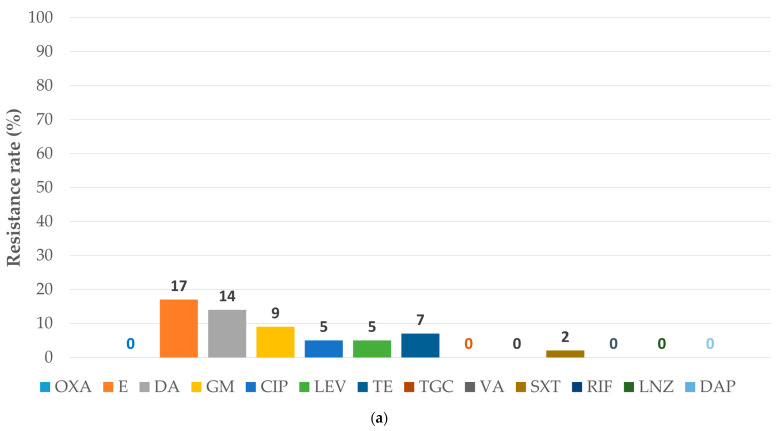

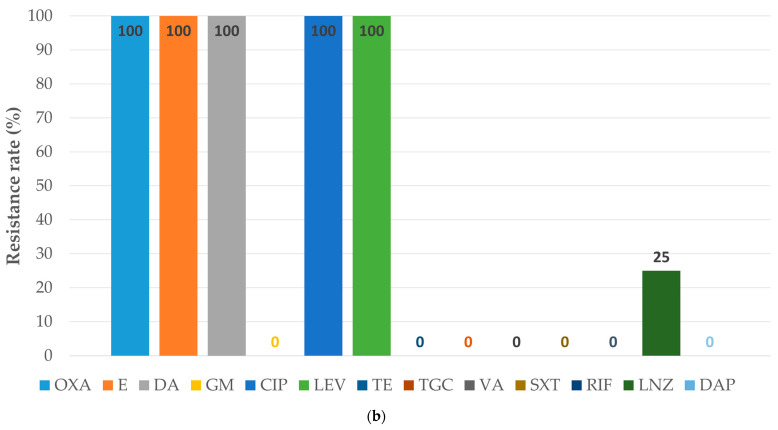
Antibiotic resistance rates in *Staphylococcus aureus*: (**a**) the subgroup sensitive to methicillin (sample size: 58); (**b**) the subgroup resistant to methicillin (sample size: 4). MRSA: methicillin-resistant *Staphylococcus aureus*; MSSA: methicillin-susceptible *Staphylococcus aureus*. The abbreviations for antibiotics are from the European Committee on Antimicrobial Susceptibility Testing Breakpoint tables for interpretation of minimum inhibitory concentration and zone diameters Version 15.0. valid from 2025-01-01 [17]: CIP: ciprofloxacin; DA: clindamycin; DAP: daptomycin; E: erythromycin; GM: gentamicin; LEV: levofloxacin; LNZ: linezolid; OXA: oxacillin; RIF: rifampicin; SXT: trimethoprim-sulfamethoxazole; TE: tetracycline; TGC: tigecycline; VA: vancomycin.

**Figure 2:**
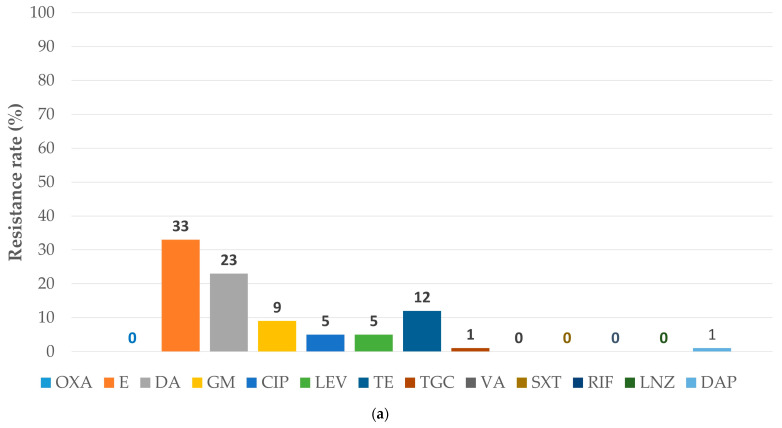

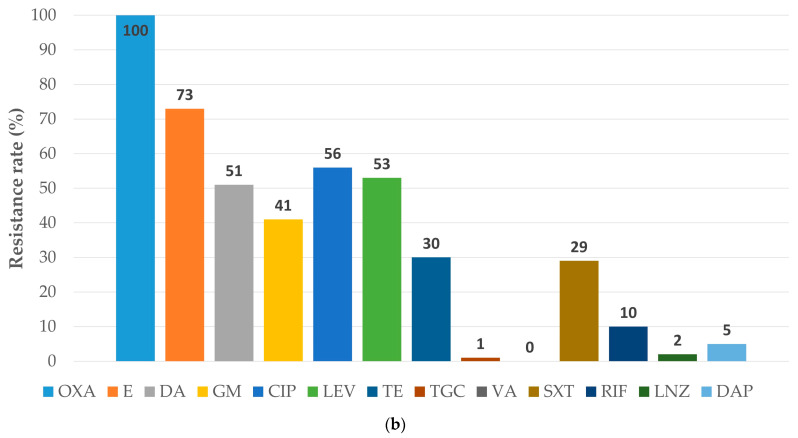
Antibiotic resistance rates in coagulase-negative staphylococci: (**a**) the subgroup sensitive to methicillin (sample size: 78); (**b**) the subgroup resistant to methicillin (sample size: 99). MR-CoNS: methicillin-resistant coagulase-negative staphylococci; MS-CoNS: methicillin-susceptible coagulase-negative staphylococci. The abbreviations for antibiotics are from the European Committee on Antimicrobial Susceptibility Testing Breakpoint tables for interpretation of minimum inhibitory concentration and zone diameters Version 15.0. valid from 2025-01-01 [17]: CIP: ciprofloxacin; DA: clindamycin; DAP: daptomycin; E: erythromycin; GM: gentamicin; LEV: levofloxacin; LNZ: linezolid; OXA: oxacillin; RIF: rifampicin; SXT: trimethoprim-sulfamethoxazole; TE: tetracycline; TGC: tigecycline; VA: vancomycin.

Figure 1 and Figure 2 compare AMR patterns for MSSA and MS-CoNS with those for MRSA and MR-CoNS.

**Figure 3:**
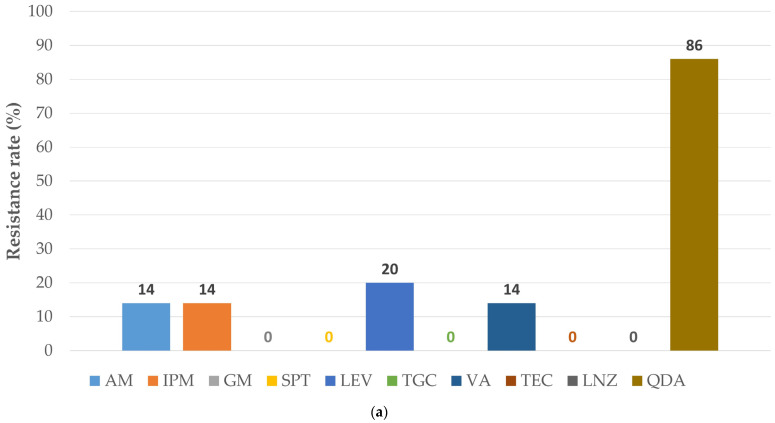

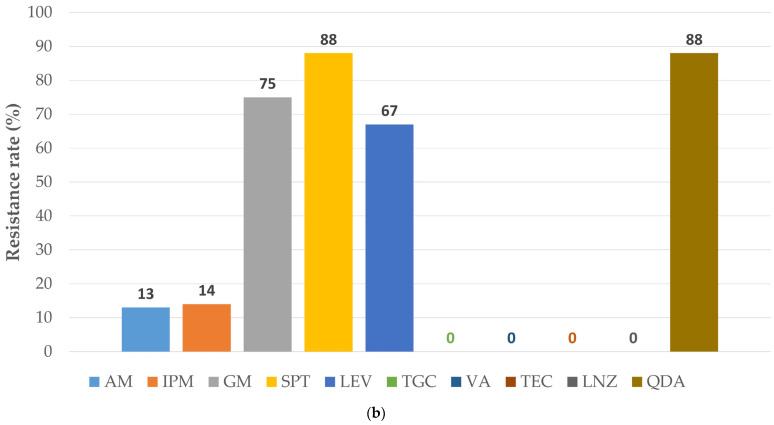
Antibiotic resistance rates in enterococci: (**a**) the subgroup sensitive to high-level aminoglycoside resistance (sample size: 7); (**b**) the subgroup resistant to high-level aminoglycoside resistance (sample size: 8). The abbreviations for antibiotics are from the European Committee on Antimicrobial Susceptibility Testing Breakpoint tables for interpretation of minimum inhibitory concentration and zone diameters Version 15.0. valid from 2025-01-01 [17]: AM: ampicillin; GM: gentamicin; IPM: imipenem; LNZ: linezolid; QDA: quinupristin/dalfopristin; SPT: spectinomycin; TEC: teicoplanin; TGC: tigecycline; VA: vancomycin.

The HLAR was observed in 53.8% of *E. faecalis* (30.8% for both gentamycin and streptomycin), along with levofloxacin co-resistance (66.7%), or intrinsic resistance to streptogramins A and B (100.0%). *E. faecalis* strains with HLAR presented a trend to higher MDR prevalence (*p* = 0.07); Supplementary Table S3. Conversely, HLAR *E. faecium* (one of two isolates, 50.0%) coexisted with resistance to ampicillin, vancomycin (vanB type), and levofloxacin but not quinupristin/dalfopristin. The VRE isolate was tigecycline- and linezolid-susceptible. Figure 3 compares AMR patterns for non-HLAR vs. HLAR enterococci.

None of the streptococcal strains met the MDR criterion. β-lactam resistance was observed only among VGS (40.0%), with co-resistance to lincosamides in 50.0%. Single isolates of GBS and group C streptococci were mainly sensitive to commonly used antibiotics.

## 3. Discussion

In the presented Silesian cohort from the “EXTRACT” registry, Gram-positive cocci were the main etiological factor of CIED-related infections, while staphylococci were the most common isolates. Among the 263 cultured Gram-positive strains, 92 (34.98%) were MDR, with the highest MDR rate observed among CoNS (46.9%). Methicillin resistance in staphylococci most often coexisted with resistance to oral antibiotics accepted for use for isolated PI, such as lincosamides, tetracyclines, and co-trimoxazole in 30% to up to 100% of cases. Importantly, all methicillin-resistant staphylococci remained susceptible to vancomycin, the recommended second-line therapy for CIED-IE. The susceptibility of MRSA/MR-CoNS to other second-line antimicrobials, such as linezolid, rifampicin, daptomycin, and tigecycline, was also high. All *E. faecalis* strains, but not those of *E. faecium*, were susceptible to ampicillin, the first-line drug for the treatment of enterococcal infections, including systemic infections. However, high-level aminoglycoside resistance—critical for combined therapy in CIED-related infective endocarditis—was observed in 53.8% of *E. faecalis* and 50% of *E. faecium*. The prevalence of VRE was 6.7%; the VRE *E. faecium* strain remained susceptible to rescue-therapy agents such as linezolid and tigecycline. Among a rare isolated streptococci, co-resistance to β-lactams and lincosamides was observed, although only in the VGS.

The discussion analyzed the collected local data on AMR/MDR in the context of standards for empirical and targeted therapy of CIED-related infections [7,22,23,24,25,26,27,28,29,30] and recent national data [3,4,5,9,10,31,32,33,34,35,36,37,38], rather than data on heterogeneous antibiotic resistance rates worldwide.

### 3.1. β-Lactam-Susceptible Gram-Positive Cocci

Treatment with β-lactam antibiotics is characterized by high potency, good tissue penetration, and low toxicity, making them safe even for elderly patients and those with chronic renal failure or other comorbidities.

Narrow-spectrum β-lactamase (penicillinase) production is common among staphylococci (100% of strains in the Silesian cohort). The enzyme inactivates benzylpenicillin, phenoxymethylpenicillin, ampicillin/amoxicillin, piperacillin, and ticarcillin by cleaving the β-lactam ring [20,21,39]. Therefore, these antibiotics are not currently used in treating staphylococcal infections.

First-line treatment for penicillinase-producing staphylococci in Poland includes cloxacillin. This semisynthetic, penicillinase-resistant, isoxazolylpenicillin has the highest activity against MSSA/MS-CoNS, although it is mainly administered intravenously due to limited oral bioavailability [7,23,25,27]. The second option involves first-generation cephalosporins [7,23,25,27,29], some of which are available as oral formulations (e.g., cefadroxil in Poland). Penicillinase-producing strains can also be treated with β-lactams combined with β-lactamase inhibitors, other cephalosporins (except cefixime, ceftazidime, ceftazidime-avibactam, ceftibuten, and ceftolozane-tazobactam), and carbapenems [27,29]. In most cases, cephalosporins can be safely used in patients with self-reported penicillin/ampicillin allergy [40]. Penicillinase production is sporadic in enterococci and has not been reported in streptococci [20,27]. Therefore, penicillin G and more commonly ampicillin/amoxicillin remain the preferred options for β-lactam-sensitive enterococci (combined with ceftriaxone or gentamicin in CIED-IE) and streptococci (except for phenoxymethylpenicillin and isoxazolylpenicillins for GBS, due to unconfirmed clinical efficacy) [23,27,28].

In the Silesian cohort, *S. aureus*, enterococci, and streptococci showed high β-lactam susceptibility: 93.5% to cloxacillin and first-generation cephalosporins for *S. aureus*, 100.0% to ampicillin/amoxicillin for *E. faecalis* (not for *E. faecium*, which was ampicillin-resistant), and 75% to penicillin and third-generation cephalosporins in VGS (100% for GBS and group C). Conversely, only 44% of CoNS were susceptible to cloxacillin/methicillin (MS-CoNS). The prevalence of β-lactam-susceptible strains remained stable from 2016 to 2025, and aligned with the national average reported by the Polish National Reference Centre for Antimicrobial Susceptibility [31].

### 3.2. β-Lactam-Resistant Gram-Positive Cocci

Most Gram-positive cocci (except for group A streptococci and the vast majority of strains in groups C and G) have developed resistance to β-lactam antibiotics by modifying penicillin-binding proteins (PBPs), leading to ineffective inhibition of bacterial cell wall synthesis [20]. In staphylococci, the most significant resistance mechanism is the synthesis of low-affinity PBP2a [21,39]. In enterococci, especially *E. faecium*, overproduction of the low-affinity PBP5 is a key factor in ampicillin resistance, while these organisms are intrinsically resistant to cephalosporins [20,27]. Altered PBP mosaic genes observed mainly in *S. pneumoniae* and VGS, resulting in varying degrees of impaired penicillin- and third-generation cephalosporin susceptibility [16,20,32].

According to data from the EARS-Net for 2024 [18], the population-weighted mean MRSA percentage was 14.2%, down 2.5% from 2020. The percentage of β-lactam-resistant *E. faecalis* is below 1%, whereas *E. faecium* approaches 100%. The population-weighted mean percentage of *S. pneumoniae* with a benzylpenicillin minimum inhibitory concentration (MIC) >0.06 mg/L (indicating susceptible, increased exposure, or penicillin-resistant strains) was 17%. Furthermore, up to 11% of strains exhibited macrolide co-resistance, and around 5–10% of isolates from invasive infections were resistant to third-generation cephalosporins [16,18]. In VGS, penicillin resistance varies among species from 9% to 23% [32], whereas the prevalence of reduced susceptibility to penicillin in GBS is about 8.5% [33].

Increased β-lactam resistance among Gram-positive cocci forces the use of more expensive, higher-toxicity antibiotics with narrow therapeutic windows (e.g., aminoglycosides, glycopeptides, oxazolidinones, tetracyclines) or those with suboptimal pharmacokinetic properties (e.g., aminoglycosides, glycopeptides) [7,23]. Antimicrobial susceptible, increased exposure strains require higher antibiotic doses or concentrations at the infection site for treatment success [23,27].

### 3.3. Resistance to Oral Non-β-Lactam Antibiotics

In environments with a high prevalence of MRSA/MR-CoNS, clindamycin offers an alternative to β-lactams for empirical oral therapy of acute bacterial skin and skin-structure infections (ABSSSI) [25]. However, the average resistance of *S. aureus* to erythromycin and clindamycin in Poland is 28% and 25%, respectively [31], most often due to macrolides/lincosamides/streptogramin B (MLS_B_) cross-resistance, associated with ribosomal target-site modification or efflux pump activity [20,21,39]. In fact, the rates of MLS_B_ resistance among MRSA/MR-CoNS can be even higher [41], with our cohort revealing that all MRSA isolates exhibited co-resistance to macrolides/lincosamides, and 34% of MR-CoNS followed suit. Other data from the Silesian Voivodeship [10] show that 12% of *S. aureus* and 59% of CoNS from bloodstream infections displayed co-resistance to both methicillin and MLS_B_.

For moderate-severity purulent ABSSSI, co-trimoxazole and doxycycline are also recommended [25]. Moreover, there are some reports of the effective use of high-dose co-trimoxazole with clindamycin as oral step-down therapy in the treatment of CIED-IE caused by MRSA [42]. However, our observations noted tetracycline resistance in 22% of CoNS (30% in MR-CoNS) and in 7% of *S. aureus* (MSSA only), and co-trimoxazole resistance in 16% of CoNS (30% of MR-CoNS) and in 1.6% of MSSA. The resistance rates for tetracycline and co-trimoxazole in MRSA strains were lower than those reported for Polish hospitals in 2005 [34].

Polish epidemiological investigations revealed stable AMR prevalence in *Streptococcus pyogenes* (7–22% for erythromycin and 5–28% for tetracycline) [35]. Macrolides/lincosamides resistance in *S. pyogenes* is clinically important for the combined treatment of streptococcal necrotizing soft tissue infections [43]. Among GBS and VGS strains, erythromycin and clindamycin resistance is observed in 25–30% of cases [32,33]. In our cohort, no *S. pyogenes* was cultured. Among GBS and VGS, lincosamide resistance was observed in 40–50% of strains, with co-resistance to β-lactams in half of the VGS strains. Isolated GBS were susceptible to ampicillin, tetracycline, and co-trimoxazole.

Macrolides, lincosamides, streptogramins (except quinupristin/dalfopristin for *E. faecium*), and tetracyclines (except tigecycline) are not used in enterococcal infections. Co-trimoxazole is also not recommended, as its activity against enterococci is uncertain [27].

Another problem is the reduced susceptibility of staphylococci to fluoroquinolones caused by mutations in target enzymes (topoisomerases) [20,21,39]. Therefore, these drugs are not recommended as monotherapy, despite the availability of oral formulations and simple dosing. Resistance to methicillin and fluoroquinolones is the most common combination reported in *S. aureus* (7.3% in EARS-Net for 2024) [18]. In our cohort, a consistently high ciprofloxacin/levofloxacin resistance rate was observed from 2016, i.e., 11% among *S. aureus* (100% among MRSA) and 33.5% among CoNS (56% among MR-CoNS). However, the newer fluoroquinolone, moxifloxacin, in combination with amoxicillin or rifampicin, is recommended for partial oral therapy of endocarditis caused by *E. faecalis* or VGS [23]. Unfortunately, susceptibility testing for enterococci and streptococci to fluoroquinolones, including moxifloxacin, was not routinely performed at our center.

### 3.4. Aminoglycoside-Resistant Enterococci and Staphylococci

In initial empirical treatment of CIED-IE, broad-spectrum intravenous combined therapy is recommended, including an antibiotic effective against MRSA/MR-CoNS (vancomycin, alt. daptomycin) and Gram-negative bacteria (third-generation cephalosporin, alt. gentamicin for patients with normal renal function) [7,23]. Combined antimicrobial therapy is also recommended for all enterococcal infections and VGS infections with “penicillin-susceptible, increased exposure” or penicillin-resistant strains (MIC 0.5–1.0 mg/L or >1.0 mg/L, respectively); it includes vancomycin and gentamicin for enterococci or a β-lactam and gentamicin for VGS [7,23,28]. Combined antibiotic therapy with gentamicin is also recommended in staphylococcal prosthetic valve endocarditis, but not in native valve endocarditis [23].

Gram-positive cocci are resistant to aminoglycosides when used alone due to poor transport across the cytoplasmic membrane. However, a synergistic effect with β-lactams or glyco- and lipopeptides (cell wall-active agents) remains possible if the isolate does not produce acquired aminoglycoside-modifying enzymes (AMEs), resulting in HLAR [44,45]. When using such a therapy regimen, it is mandatory to take into account that both vancomycin and aminoglycosides have narrow therapeutic windows due to nephrotoxicity. To reduce complication risk, monitoring of renal function, and controlling the minimum and maximum concentrations for aminoglycosides (pre-dose concentrations < 1 mg/L and post-dose [peak; 1 h after injection] serum concentrations 10–12 mg/L) and the 24 h area under the concentration-time curve-to-minimum inhibitory concentration ratio (AUC24/MIC 400–600 mg·h/L) for vancomycin are required [46,47]. The dosage of gentamicin in combined therapy for CIED-IE is lower than in monotherapy for urinary tract infections (i.e., 3 mg/kg/day using lean body weight, administered in a single dose, maximum dose 240 mg/day), and the treatment duration should be shortened to 2 weeks [23].

Unfortunately, in 2024, the European population-weighted mean percentage of HLAR was 22.6% in *E. faecalis* and 16.5% in *E. faecium* [18]. According to Polish data [31], the HLAR rate for gentamicin is higher: 55.2% for *E. faecalis* and 50.2% for *E. faecium*. The Silesian cohort presented similarly high HLAR prevalence, up to 40.0% of cases for gentamycin. Isolates of enterococci with HLAR for gentamicin may still exhibit synergistic activity with streptomycin [27]. However, in the Silesian cohort, 30.8.% of *E. faecalis* HLAR and 50.0% of *E. faecium* HLAR were simultaneously resistant to both of these aminoglycosides. Moreover, HLAR *E. faecium* were co-resistant to ampicillin in one of two cases.

In Silesia, AMEs were also detected in 9% of *S. aureus*, only in MSSA strains. This percentage is higher than the national average in Poland, which is 3.9% [31]. In CoNS, AMEs prevalence was at 30%, higher in the MR-CoNS subgroup, up to 40%. Gentamicin-resistant staphylococci, by producing the two-domain acetyltransferase/phosphotransferase AAC(60)-Ie/APH(2”)-Ia, are usually resistant to other aminoglycosides, i.e., tobramycin, kanamycin, amikacin, and netilmicin [44].

### 3.5. Vancomycin-Resistant Gram-Positive Cocci

Glycopeptide antibiotics are the drugs of choice for severe infections caused by β-lactam-resistant Gram-positive cocci. They act by inhibiting peptidoglycan synthesis. In VRE and VRSA, inducible resistance to high glycopeptide concentrations (MICs to vancomycin 64–1024 mg/L) is conferred by the VanA operon, resulting in the synthesis of modified cell wall precursors with low affinity for vancomycin and teicoplanin [21,39]. However, among enterococci, other glycopeptide resistance phenotype is VanB, characterized by inducible resistance to vancomycin with retained susceptibility to teicoplanin [48]. In staphylococci, resistance variants with a van-independent mechanism (previously known as vancomycin-intermediate *S. aureus*, VISA) are much more prevalent. They are characterized by a relatively low vancomycin MIC of 4–8 mg/L, and are determined by multiple genes that alter the bacterial cell wall [36]. Regardless of these differences, in cases with a vancomycin MIC >1 mg/L, alternative therapy is recommended, such as daptomycin (or newer-generation lipopeptide antibiotics) in combination with cloxacillin, ceftaroline (a cephalosporin with high affinity for PBP2a found in MRSA strains), or fosfomycin. *S. aureus* strains with vancomycin MIC of 2 mg/L (at the border of wild-type distribution) should be considered vancomycin-resistant [23,27,28]. Glycopeptide-resistant streptococci groups A, B, C, G, and VGS are rare or have not yet been reported, as is *S. pneumoniae* [20,27].

In recent years, the rate of VRSA in Europe increased from 0.58% to 2.21% among both hospital and community isolates [18]. Over the period 1991–2007, in Warsaw hospitals (Poland), among 600 MRSA strains, only 47 were resistant to glycopeptides. All exhibited van-independent mechanisms of resistance [36]. In 2024, the State Sanitary Inspectorate registered 211 VRSA infections nationwide [37]. Fortunately, in the Silesian cohort, no VRSA were cultured.

In turn, the European population-weighted average percentage of *E. faecium* resistant to vancomycin was slightly lower in 2024 than in 2020 (currently 16.5%). However, there was an increase in bloodstream infections caused by VRE *E. faecium* (currently 1.96 per 100,000 population) [18]. Unfortunately, the high rates of resistance of *E. faecium* to vancomycin (34.3%) and teicoplanin (28.0%) place Poland among the countries with the highest percentages of vancomycin-resistant *E. faecium* strains in Europe [31]. It is confirmed by data from the University Teaching Hospital in Bialystok (Poland, Podlaskie Voivodeship) [38]. There, *E. faecium* VRE was the dominant MDR pathogen, with an incidence of 11.4 per 1000 hospitalized patients. According to data from a large microbiological laboratory serving the Silesian Voivodeship [10], *E. faecium* with combined VRE and HLAR was found in 17.1% of bloodstream infections, with a frequency of up to 50% in surgical units. In our cohort, one teicoplanin-resistant (VanB type) *E. faecium* strain was found. Glycopeptide resistance is independent of β-lactam resistance, and some VRE strains may retain ampicillin susceptibility [27], but this was not observed in our cohort. Concerning *E. faecalis*, glycopeptide resistance is less common in Poland, with vancomycin resistance rates of 4.4% and teicoplanin resistance rates of 3.2% [31].

### 3.6. Susceptibility to Salvage-Therapy Antibiotics in MDR Gram-Positive Cocci

In cases of resistance to first- and second-line therapy, treatment should be based on linezolid, tigecycline, or daptomycin (or newer lipopeptides), all dosed parenterally, often in combination with other antimicrobials [24,49,50,51,52]. Unfortunately, simultaneous resistance of MRSA, VRSA, and VRE strains to antimicrobials used as salvage therapy is increasingly observed [13,49,50,51,52].

The oxazolidinones are active against MDR cocci, including MRSA, VISA, VRSA, and VRE [21,49]. The percentage of linezolid-resistant strains is relatively low: 1.4% for staphylococci and 0.3% for enterococci in Poland [31,37]; 1.6% for *S. aureus* (higher for MRSA, at 25.0%), 1.1% for CoNS (2.0% for MR-CoNS), and no linezolid-resistant strains among enterococci or streptococci in the Silesian cohort. The low prevalence of linezolid resistance supports the use of oral formulation in outpatient settings, as an acceptable treatment option for CIED-IE caused by MR-CoNS, *E. faecalis*, and VGS [13,23]. However, in Poland, prolonged oral linezolid therapy is not reimbursed and is considered prohibitively expensive.

High-dose daptomycin (≥8 mg/kg/day) can be effective in the treatment of bloodstream infections and endocarditis caused by MRSA, MR-CoNS, and VRE when combined with other agents [30,49,50]. The prevalence of daptomycin resistance among staphylococci is low [13,30]; in the Silesian cohort, resistant strains were only among CoNS, at a rate of 3.5%. Dalbavancin acts against VISA and vanB-type VRE [49,51,52]. However, cases of vancomycin and dalbavancin resistance induction during prolonged treatment have been reported in patients with central line-associated bloodstream infections and CIED-IE [53,54]. In comparison, oritavancin has a broader spectrum of activity, covering VRSA and vanA-type VRE, and its use is safe and acceptable in long-term therapy [24,50,51,52,55]. In 2010–2019, of 15,403 Gram-positive bacterial isolates from blood cultures in the United States, 99.5% of *S. aureus*, 98.4–100.0% of CoNS, 96.2–99.1% *E. faecalis*, and 93.8–100.0% of VGS isolates were oritavancin-susceptible [56]. Susceptibility to semisynthetic lipopeptides is not routinely assessed. In clinical practice, confirmed vancomycin susceptibility implies susceptibility to dalbavancin and/or oritavancin [27,52]. In patients with MDR CIED-IE, outpatient parenteral antimicrobial therapy with long-acting lipopeptides is a treatment option worth considering [23,24].

Rifampicin is a bactericidal antibiotic considered for oral step-down therapy in cases with staphylococcal or VGS CIED-IE or prosthetic valve endocarditis [23,27,28]. However, EARS-Net for 2024 reports 0.5% co-resistance to rifampicin in MRSA strains [18]. In our cohort, 9.6% of MR-CoNS were rifampicin-resistant, but none of the MRSA were.

Tigecycline, a semisynthetic tetracycline, is a broad-spectrum bacteriostatic agent with activity against Gram-positive and Gram-negative organisms. Tigecycline provides an alternative treatment for MRSA, VRE, penicillin-resistant *S. pneumoniae*, and mixed infections with Gram-negative bacteria in complicated ABSSSI. However, due to its relatively low serum concentrations, it should be avoided in the treatment of systemic infections [49,51,57]. In Europe, tigecycline resistance in Gram-positive cocci isolates is rarely reported [57]. Similarly, in the Silesian cohort, tigecycline-resistant strains were cultured only among CoNS, at a rate of 1.1%.

Streptogramin A (dalfopristin) and streptogramin B (quinupristin) given together can have a bactericidal effect against many Gram-positive cocci, including MRSA, VRSA, and VRE (only *E. faecium*) [13,20]. However, the drug was withdrawn from production in 2022 due to its significant side effects and drug–drug interactions.

### 3.7. Multidrug Resistance in CoNS

The higher resistance rates observed among CoNS compared to other Gram-positive cocci are related to the biological and epidemiological properties of this group of microorganisms. CoNS are common commensals of human skin and mucosal surfaces and are therefore constantly exposed to antimicrobial agents used both in hospital and community settings. This promotes strong selective pressure for the emergence of antibiotic-resistant strains [58]. Furthermore, these bacteria demonstrate a remarkable ability to acquire and accumulate antimicrobial resistance determinants through horizontal gene transfer. These include genes such as mecA, associated with resistance to β-lactam antibiotics, erm genes, associated with resistance to macrolides, lincosamides, and streptogramins, and aminoglycoside-modifying enzymes. CoNS are also recognized as major reservoirs of mobile genetic elements, such as SCCmec cassettes, which can be transferred between staphylococcal species and contribute to the spread of resistance in the hospital environment [59,60]. Another important factor contributing to increased resistance is their marked ability to form biofilms on indwelling medical devices, such as catheters and prosthetic materials, compared with other Gram-positive microorganisms. Biofilm formation significantly limits the penetration of antimicrobial drugs into the biofilm structure and promotes the persistence of bacterial populations, increasing antibiotic tolerance. CoNS are increasingly among the main pathogens in healthcare settings, where intensive antibiotic use further drives the selection and persistence of MDR strains [61,62].

### 3.8. Study Limitations

We presented the results of a single-center study conducted in the Silesian Voivodeship (Poland). Regional variation in AMR rate is well known. Therefore, the study’s results may be most relevant to local centers and can be extended to Poland, given the compatibility of the AMR rates with national data. However, they should be considered with caution in other countries. Moreover, the AMR rate may change over time, but this was not the case in the Silesian cohort from 2016 to 2025.

Periprocedural procedures, including the types and quantities of microbiological samples collected at the center, remained constant throughout the study period. Furthermore, the researchers had full access to the registry database and source medical records, which helped reduce missing data.

Another limitation is the small number of isolates of streptococci and enterococci, reflecting their significantly lower prevalence among the etiological agents of CIED-related infections, accounting for only a few percent of cases [3,9]. A small sample size in that subgroup reduces data reliability. However, data on drug resistance in these pathogens among patients with CIED infections in the Polish population are scarce. Furthermore, these pathogens predominantly cause systemic infections, including bacteremia and infective endocarditis [3,9,10]. Thus, we believe the Silesian cohort data will be clinically relevant for comparative assessment and meta-analysis.

Additionally, CoNS are constantly present on the skin of patients, staff, and in the hospital environment. Therefore, in cases where CoNS were cultured from the pocket fistula/erosion, single sample, or among multiple bacterial species, it was difficult to definitively conclude whether they are the true etiological factors in CIED infection [62]. These strains were rarely excluded due to concerns about delaying or neglecting treatment for systemic infections, potentially leading to an overestimation of CoNS prevalence.

Susceptibility to some antimicrobials used only in combined therapy, e.g., fosfomycin and fusidic acid, is not routinely assessed in our center, and we considered their discussion to be beyond the scope of this manuscript.

## 4. Materials and Methods

The research data were presented in accordance with STROBE (STrengthening the Reporting of OBservational studies in Epidemiology) and RECORD (REporting of studies Conducted using Observational Routinely collected Data) guidelines for studies conducted using routinely collected health data [63].

### 4.1. Study Group

All data come from the prospective TLE registry “EXTRACT” (Effectiveness, Complications, and Mortality of TLE in Patients; ClinicalTrials.gov ID: NCT05775783), conducted at the Department of Electrocardiology and Heart Failure of the Medical University of Silesia in Katowice, Poland. The registry included 702 consecutive patients who underwent at least 1 lead extraction (implanted at least 1 year earlier) and were treated between January 2016 and June 2025. The study group (209 patients) consisted of “EXTRACT” participants aged ≥18, both sexes, undergoing the TLE procedure due to diagnosis of CIED-related infection, in accordance with the European Heart Rhythm Association [7] and the European Society of Cardiology [23] guidelines. Definitions of CIED-related infection types were added in Supplementary Table S1.

### 4.2. Data Extraction and Quality Assessment

Researchers had full access to the population database used to create the study population. Independent researchers (D.L., S.G.-W., M.J. and J.S.) extracted and verified data from the “EXTRACT” registry. It included the following information: cohort characteristics (including anthropometric data and comorbidities), clinical data (including indications for the TLE procedure, laboratory data, and echocardiographic test results), and microbiological data (including the type of sample collected, the type of strain cultured, and strain antibiotic susceptibility).

Quality assessment involved checking for duplicate entries, verifying internal consistency (e.g., dates, infection type, microbiological results), and ensuring completeness of key variables. Missing or implausible values were reviewed and corrected when possible. Cases with negative cultures, comprising 6.2% (13 patients), were not excluded from the study group. Qualitative susceptibility categories of “sensitive, standard dosing regimen”, “sensitive, increased exposure”, and “resistant” were applied according to the European Committee on Antimicrobial Susceptibility Testing (EUCAST) criteria.

### 4.3. Operating and Microbiological Procedures

TLE procedures were performed by an experienced extraction team in the hybrid catheterization laboratory operating room, according to the expert consensus statements on lead extraction [64], as previously described [65,66].

To minimize false-positive microbial cultures due to skin flora, intraoperative cultures from surgical wounds and surgically extracted endocardial leads (approximately four centimeters long) were collected aseptically. At least three peripheral blood samples were collected from each patient for microbiological culture under aerobic and anaerobic conditions. Swabs from the pocket fistula/erosion were collected only as auxiliary materials. Sole isolates from fistula or generator pocket erosions were considered etiological factors when other cultures were negative; these accounted for 11.4% of cases.

Bacterial cultures and drug susceptibility testing were performed according to standard procedures, consistent with the European Society of Clinical Microbiology and Infectious Diseases and EUCAST guidelines [22,27,28]. Preliminary analysis was performed using the fully automated microbiology identification system VITEK 2 (bioMérieux SA, Marcy-l’Etoile, France) supported by the Advanced Expert System (AES™) software (bioMérieux, Saint Louis, MO, USA). The AES™ software identifies resistance mechanisms based on a phenotypic assessment of bacterial resistance patterns, including growth curve analysis in the presence of antibiotics, MICs, resistance synergisms, and atypical resistance phenotypes [67,68]. Results were manually verified using the disk diffusion method. Qualitative susceptibility categories of “sensitive, standard dosing regimen”, “sensitive, increased exposure”, and “resistant” were applied according to breakpoint criteria [17,24]. Strains that are resistant to three or more antimicrobial classes were defined as MDR [69].

### 4.4. Statistical Analysis

Results were analyzed using MedCalc Version 23.3.7 (MedCalc Software Ltd., Ostend, Belgium). Continuous variables were reported as mean and SD or median with Q1–Q3 quartiles, depending on the distribution’s normality, assessed by the Kolmogorov–Smirnov test. Quantitative variables were analyzed as continuous variables. Univariate analysis of independent variables was carried out using ANOVA with the Tukey–Kramer post hoc test or the Kruskal–Wallis rank test with Conover’s post hoc test, as required. Qualitative data were presented as counts and percentages and compared using the chi-square test or Fisher’s exact test, depending on the sample size. The year of procedure (treated as a categorical variable, the Cochran–Armitage trend test) was used to assess temporal trends in AMR prevalence. A two-sided *p*-value < 0.05 was considered statistically significant.

## 5. Conclusions

Multidrug resistance was observed predominantly among CoNS (especially methicillin-resistant strains), MRSA, and HLAR enterococci. It significantly limits therapeutic options for CIED-related infections, including combined therapy and oral step-down therapy. Local epidemiological data can help optimize the initial empiric therapy.

## Figures and Tables

**Table 1:** Baseline characteristics of the study group by the infection type.

Data	All Patients	Group I (Isolated PI)	Group II (PI with Bacteriemia/CIED-IE)	Group III (Isolated Bacteremia/CIED-IE)	*p*
	n 209	n 107	n 55	n 47	
**General characteristics**					
Age (years), M (SD)	66.24 (12.99)	71.36 (13.15)	72.15 (10.40)	68.30 (9.84)	0.22
Sex (male), n (%)	153 (73.2)	79 (51.6)	42 (27.5)	32 (20.9)	0.63
BMI (kg/m^2^), M (SD)	28.44 (5.12)	27.45 (4.20)	28.72 (5.21)	28.48 (4.14)	0.21
**Laboratory parameters**					
Hemoglobin * (g/dL), M (SD)	13.52 (2.07)	13.28 (1.87)	12.59 (2.09)	10.69 (2.20)	<0.001 I ≠ III, II ≠ III
WBC * (cells/mL), M (SD)	7.67 (2.68)	7.76 (3.35)	8.77 (3.30)	9.38 (4.00)	0.02 I ≠ III
PLT * (cells/mL), M (SD)	213.05 (79.78)	212.50 (72.12)	221.84 (110.68)	228.45 (124.89)	0.62
C-reactive protein * (mg/L), Me (Q1-Q3)	2.50 (2.50–8.60)	2.50 (2.50–13.30)	11.30 (2.50–87.58)	35.40 (11.50–100.00)	<0.001 I ≠ II, I ≠ III, II ≠ II
Creatinine * (mg/dL), M (SD)	1.12 (0.61)	1.26 (0.90)	1.20 (0.46)	1.48 (0.93)	0.19
**Comorbidities**					
Coronary artery disease, n (%)	123 (58.9)	62 (50.4)	34 (27.6)	27 (22.0)	0.87
Hypertension, n (%)	156 (74.6)	84 (53.8)	46 (29.5)	26 (16.7)	0.002
Congestive heart failure ^a^, n (%)	118 (56.5)	67 (56.8)	27 (22.9)	24 (20.3)	0.18
Atrial fibrillation, n (%)	99 (47.4)	52 (52.5)	26 (26.3)	21 (21.2)	0.90
Diabetes mellitus, n (%)	79 (37.8)	39 (49.4)	20 (25.3)	20 (25.3)	0.75
Chronic kidney disease ^b^, n (%)	102 (48.8)	51 (50.0)	26 (25.5)	25 (24.5)	0.79
Stroke, n (%)	20 (9.6)	12 (60.0)	5 (25.0)	3 (15.0)	0.64
Peripheral artery disease, n (%)	83 (39.7)	41 (49.4)	26 (31.3)	16 (19.3)	0.36
Chronic obstructive pulmonary disease, n (%)	28 (13.4)	14 (50.0)	8 (28.6)	6 (21.4)	0.96
Cancer, n (%)	17 (8.1)	11 (64.7)	2 (11.8)	4 (23.5)	0.34
**Cardiac implantable electronic device type**					
Pacemaker, n (%)	100 (47.8)	47 (47.0)	28 (28.0)	25 (25.0)	0.83
Implantable cardioverter- defibrillator, n (%)	55 (26.3)	31 (56.4)	13 (23.6)	11 (20.0)	
Cardiac resynchronization therapy–pacemaker/defibrillator, n (%)	54 (25.8)	29 (53.7)	14 (25.9)	11 (20.4)	

BMI: body mass index; CIED-IE: cardiac implantable electronic device-related infective endocarditis; n: number (sample size); PLT: platelet count; PI: pocket infection/erosion; WBC: white blood cell count. * The range of laboratory standards used in the center: Hemoglobin 13.7–16.5 (g/dL); WBC 3.90–9.50 (cells/mL); PLT 135–350 (cells/mL); C-reactive protein < 5.0 mg/L; Creatinine 0.67–1.17 mg/d. ^a^ Congestive heart failure with left ventricular ejection fraction ≤ 50%, ^b^ Chronic kidney disease was defined as estimated glomerular filtration rate < 60 mL/min/1.73 m^2^ calculated using the Modification of Diet in Renal Disease method. The sign ≠ denotes a difference between subgroups, with *p* < 0.05 in post hoc test.

**Table 2:** Isolated Gram-positive cocci according to the infection type.

All Strains	All Patients	Isolated PI (Group I)	PI with Bacteriemia/CIED-IE (Group II)	Isolated Bacteremia/CIED-IE (Group III)	*p* Value
	n 209	n 107	n 55	n 47	
*Staphylococcus aureus*, n (%)	62 (23.6)	34 (54.8)	17 (27.4)	11 (17.7)	0.001 I ≠ II
CoNS, n (%)	177 (67.3)	91 (51.4)	66 (37.3)	20 (11.3)	<0.001 I ≠ II, II ≠ III
*Streptococcus* spp., n (%)	8 (3.0)	2 (40.0)	1 (20.0)	2 (40.0)	0.82
*Enterococcus* spp., n (%)	15 (5.7)	3 (20.0)	6 (40.0)	6 (40.0)	0.55
Other Gram-positive cocci, n (%)	1 (0.4)	1 (100.0)	0 (0.0)	0 (0.0)	>0.999

CIED-IE: cardiac implantable electronic device-related infective endocarditis; n: number (sample size); PI: pocket infection/erosion; CoNS: coagulase-negative staphylococci; The sign ≠ denotes a difference between subgroups, with *p* < 0.05 in post hoc test.

**Table 3:** Drug susceptibility of all cultured *Staphylococcus aureus* strains and coagulase-negative staphylococci.

Species		P	OXA	E	DA	GM	CIP	LEV	TE	TGC	VA	SXT	RIF	LNZ	DAP	MDR
*S. aureus*, n (%)	S	0 (0.0)	58 (93.5)	48 (77.4)	50 (80.6)	57 (91.9)	27 (43.5)	27 (43.5)	53 (91.4)	62 (100.0)	62 (100.0)	61 (98.4)	62 (100.0)	61 (98.4)	62 (100.0)	5 (8.1)
	I	0 (0.0)	0 (0.0)	0 (0.0)	0 (0.0)	0 (0.0)	28 (45.2)	28 (45.2)	1 (1.7)	0 (0.0)	0 (0.0)	0 (0.0)	0 (0.0)	0 (0.0)	0 (0.0)	
	R	62 (100.0)	4 (6.5)	14 (22.6)	12 (19.4)	5 (8.1)	7 (11.3)	7 (11.3)	4 (6.9)	0 (0.0)	0 (0.0)	1 (1.6)	0 (0.0)	1 (1.6)	0 (0.0)	
CoNS, n (%)	S	0 (0.0)	78 (44.1)	78 (44.8)	105 (59.7)	129 (72.9)	63 (35.8)	64 (36.6)	85 (48.3)	173 (98.9)	177 (100.0)	130 (73.4)	162 (94.7)	175 (98.9)	164 (96.5)	82 (46.9)
	I	0 (0.0)	0 (0.0)	0 (0.0)	3 (1.7)	0 (0.0)	54 (30.7)	56 (32.0)	53 (30.1)	0 (0.0)	0 (0.0)	18 (10.2)	0 (0.0)	0 (0.0)	0 (0.0)	
	R	177 (100.0)	99 (55.9)	96 (55.2)	68 (38.6)	48 (27.1)	59 (33.5)	55 (31.4)	38 (21.6)	2 (1.1)	0 (0.0)	29 (16.4)	9 (5.3)	2 (1.1)	6 (3.5)	

CoNS: coagulase-negative staphylococci; I: susceptible, increased exposure; MDR: multidrug-resistant; n: number (sample size); R: resistant; S: susceptible, standard dosing regimen. The abbreviations for antibiotics are from the European Committee on Antimicrobial Susceptibility Testing Breakpoint tables for interpretation of minimum inhibitory concentration and zone diameters Version 15.0. valid from 2025-01-01 [17]: CIP: ciprofloxacin; DA: clindamycin; DAP: daptomycin; E: erythromycin; GM: gentamicin; LEV: levofloxacin; LNZ: linezolid; OXA: oxacillin; P: penicillin; RIF: rifampicin; SXT: trimethoprim-sulfamethoxazole; TE: tetracycline; TGC: tigecycline; VA: vancomycin.

**Table 4:** Drug susceptibility of all cultured streptococci by group.

Species		P	AM	CTX	CRO	DA	VA	TE	SXT	LNZ	MDR
*Streptococcus* spp. group B, n (%)	S	2 (100.0)	2 (100.0)	2 (100.0)	2 (100.0)	1 (50.0)	2 (100.0)	2 (100.0)	2 (100.0)	2 (100.0)	0 (0.0)
	I	0 (0.0)	0 (0.0)	0 (0.0)	0 (0.0)	0 (0.0)	0 (0.0)	0 (0.0)	0 (0.0)	0 (0.0)	
	R	0 (0.0)	0 (0.0)	0 (0.0)	0 (0.0)	1 (50.0)	0 (0.0)	0 (0.0)	0 (0.0)	0 (0.0)	
*Streptococcus* spp. group C, n (%)	S	1 (100.0)	1 (100.0)	1 (100.0)	1 (100.0)	1 (100.0)	1 (100.0)	not marked			0 (0.0)
	I	0 (0.0)	0 (0.0)	0 (0.0)	0 (0.0)	0 (0.0)	0 (0.0)				
	R	0 (0.0)	0 (0.0)	0 (0.0)	0 (0.0)	0 (0.0)	0 (0.0)				
*Streptococcus viridans* group, n (%)	S	3 (60.0)	3 (60.0)	3 (60.0)	3 (60.0)	3 (60.0)	4 (100.0)	not marked			0 (0.0)
	I	1 (20.0)	0 (0.0)	0 (0.0)	0 (0.0)	0 (0.0)	0 (0.0)				
	R	1 (20.0)	2 (40.0)	2 (40.0)	2 (40.0)	2 (40.0)	0 (0.0)				

I: susceptible, increased exposure; MDR: multidrug-resistant; R: resistant; S: susceptible, standard dosing regimen. The abbreviations for antibiotics are from the European Committee on Antimicrobial Susceptibility Testing Breakpoint tables for interpretation of minimum inhibitory concentration and zone diameters Version 15.0. valid from 2025-01-01 [17]: AM: ampicillin; CRO: ceftriaxone; CTX: cefotaxime; DA: clindamycin; LNZ: linezolid; P: penicillin; SXT: trimethoprim-sulfamethoxazole; TE: tetracycline; VA: vancomycin.

**Table 5:** Drug susceptibility of all cultured enterococci.

Species		AM	IPM	GM	SPT	LEV	TGC	VAN	TEC	LNZ	QDA	MDR
*E. faecalis*, n (%)	S	13 (100.0)	10 (83.3)	8 (61.5)	7 (53.8)	6 (10.0)	13 (100.0)	13 (100.0)	13 (100.0)	12 (100.0)	0 (0.0)	4 (30.8)
	I	0 (0.0)	2 (16.7)	0 (0.0)	0 (0.0)	0 (0.0)	0 (0.0)	0 (0.0)	0 (0.0)	0 (0.0)	0 (0.0)	
	R	0 (0.0)	0 (0.0)	5 (38.5)	6 (46.2)	4 (40.0)	0 (0.0)	0 (0.0)	0 (0.0)	0 (0.0)	13 (100.0)	
*E. faecium*, n (%)	S	0 (0.0)	0 (0.0)	1 (50.0)	1 (50.0)	0 (0.0)	2 (100.0)	1 (50.0)	2 (100.0)	2 (100.0)	2 (100.0)	1 (50.0)
	I	0 (0.0)	0 (0.0)	0 (0.0)	0 (0.0)	0 (0.0)	0 (0.0)	0 (0.0)	0 (0.0)	0 (0.0)	0 (0.0)	
	R	2 (100.0)	2 (100.0)	1 (50.0)	1 (50.0)	1 (100.0)	0 (0.0)	1 (50.0)	0 (0.0)	0 (0.0)	0 (0.0)	

I: susceptible, increased exposure; MDR: multidrug-resistant; R: resistant; S: susceptible, standard dosing regimen. The abbreviations for antibiotics are from the European Committee on Antimicrobial Susceptibility Testing Breakpoint tables for interpretation of minimum inhibitory concentration and zone diameters Version 15.0. valid from 2025-01-01 [17]: AM: ampicillin; GM: gentamicin; IPM: imipenem; LNZ: linezolid; QDA: quinupristin/dalfopristin; SPT: spectinomycin; TEC: teicoplanin; TGC: tigecycline; VA: vancomycin.

**Table 6:** Resistance phenotypes identified in Gram-positive cocci.

Species	Evaluation Method	Resistance Phenotype
*Staphylococcus* spp.	Growth in the presence of penicillin	Resistance phenotype associated with the production of penicillinase
	Elevated oxacillin/cefoxitin MICs	Methicillin resistance phenotype associated with changes in PBPs
	Erythromycin/clindamycin resistance patterns (synergism or lack thereof)	a. inducible or constitutive MLS_B_ cross-resistance phenotype b. isolated macrolide resistance phenotype associated with the efflux pump c. isolated lincosamide resistance phenotype
	Elevated tetracycline MICs	Resistance phenotype associated with the efflux pump
	Growth at high linezolid concentrations	High-level linezolid resistance phenotype
	High gentamicin/tobramycin MICs	HLAR phenotype associated with the enzyme AAC(6′)-Ie-APH(2″)-Ia
	Elevated ciprofloxacin/levofloxacin MICs	Fluoroquinolone resistance phenotype
	High daptomycin MIC	Reduced daptomycin susceptibility phenotype
*Enterococcus* spp.	Elevated ampicillin/penicillin MIC	Resistance phenotype associated with changes in PBPs.
	Vancomycin/teicoplanin resistance pattern	VanB-type resistance phenotype
	High MIC of gentamicin/streptomycin	HLAR phenotype associated with aminoglycoside-modifying enzymes: a. for gentamicin: AAC(6′)-Ie-APH(2″)-Ia b. for streptomycin: ANT(6)-Ia, APH(3′)-IIIa
	Increased MIC of ciprofloxacin/levofloxacin	Fluoroquinolones resistance phenotype
*Streptococcus* spp.	Elevated penicillin/cefotaxime/ceftriaxone MICs	Resistance phenotype associated with changes in PBPs
	Erythromycin/clindamycin resistance patterns (synergism or lack thereof)	Inducible or constitutive MLS_B_ phenotype
	Elevated tetracycline MICs	Resistance phenotype associated with efflux pumps or ribosomal protection proteins

AAC(6′)-Ie: Aminoglycoside 6′-N-acetyltransferase type Ie; APH(2″)-Ia: Aminoglycoside 2″-O-phosphotransferase type Ia; ANT(6)-Ia: Aminoglycoside 6-O-nucleotidyltransferase type Ia; APH(3′)-IIIa: Aminoglycoside 3′-O-phosphotransferase type IIIa; HLAR: High-level aminoglycoside-resistance; MIC: Minimum inhibitory concentration; MLS_B_: macrolides/lincosamides/streptogramin B cross-resistance phenotype; PBP: Penicillin-binding proteins.

## Data Availability

Data come from the prospective TLE registry “EXTRACT” (Effectiveness, Complications, and Mortality of TLE in Patients; ClinicalTrials.gov ID: NCT05775783). The raw data supporting the study’s conclusions will be openly available in the Polish Platform of Medical Research under the CC BY license; https://ppm.sum.edu.pl/info/researchdata/SUMe8eb11fa92a74b46a0a76136bcb6b28b/; URN: umed-kat-prod:SUMe8eb11fa92a74b46a0a76136bcb6b28b (accessed on 23 March 2026).

## Supplementary Materials

The following supporting information can be downloaded at: https://www.mdpi.com/article/10.3390/antibiotics15040345/s1, Figure S1: (a) Methicillin resistance rate in *Staphylococcus aureus* from 2016 to 2025, (b) Methicillin resistance rate in coagulase-negative staphylococci from 2016 to 2025, (c) Ampicillin resistance rate in enterococci from 2016 to 2025, (d) Penicillin resistance rate in streptococci from 2016 to 2025.; Figure S2: (a) Macrolides resistance rate in *Staphylococcus aureus* from 2016 to 2025, (b) Lincosamides resistance rate in *Staphylococcus aureus* from 2016 to 2025, (c) Macrolides resistance rate in coagulase-negative staphylococci from 2016 to 2025, (d) Lincosamides resistance rate in coagulase-negative staphylococci from 2016 to 2025.; Figure S3: The prevalence of resistance to fluoroquinolones during the observation period from 2016 to 2025: (a) Fluoroquinolones resistance rate in *Staphylococcus aureus* from 2016 to 2025s, (b) Fluoroquinolones resistance rate in coagulase-negative staphylococci from 2016 to 2025.; Figure S4: The high-level aminoglycoside resistance rate in enterococci from 2016 to 2025; Table S1: Definitions of cardiac implantable electronic device-related infection types; Table S2: Resistance patterns in methicillin-susceptible and methicillin-resistant staphylococci; Table S3: Resistance patterns in enterococci with or without a high-level aminoglycoside resistance; Table S4: The RECORD statement–checklist of items, extended from the STROBE statement, that should be reported in observational studies using routinely collected health data.

## Abbreviations

The following abbreviations are used in this manuscript:

ABSSSI	Acute bacterial skin and skin-structure infection
AMEs	Aminoglycoside-modifying enzymes
AMR	Antimicrobial resistance
CIED	Cardiac implantable electronic device
CIED-IE	Cardiac implantable electronic device-related infectious endocarditis
CoNS	Coagulase-negative staphylococci
EARS-Net	European Antimicrobial Resistance Surveillance Network
EUCAST	European Committee on Antimicrobial Susceptibility Testing
EXTRACT	“Effectiveness, Complications, and Mortality of TLE in Patients” registry
GBS	Group B streptococci
HLAR	High-level aminoglycoside-resistance
MDR	Multidrug resistance
MIC	Minimum inhibitory concentration
MLS_B_	Macrolides/lincosamides/streptogramin B cross-resistance
MR-CoNS	Methicillin-resistant coagulase-negative staphylococci
MRSA	Methicillin-resistant *Staphylococcus aureus*
MS-CoNS	Methicillin-susceptible coagulase-negative staphylococci
MSSA	Methicillin-susceptible *Staphylococcus aureus*
PBPs	Penicillin-binding proteins
PI	Pocket infection/pocket erosion
TLE	Transvenous lead extraction
VGS	Viridans group streptococci
VISA	Vancomycin-intermediate *Staphylococcus aureus*
VRE	Vancomycin-resistant enterococci
VRSA	Vancomycin-resistant *Staphylococcus aureus*
