# Supplementary material for: Drug-Resistant Gram-Positive Cocci as Etiological Factors of Cardiac Implantable Electronic Device Infections—Data from the EXTRACT Registry
Source: Antibiotics (Basel). 2026 Mar 27;15(4):345. doi: 10.3390/antibiotics15040345 (PMC13113275; doi:10.3390/antibiotics15040345)
Supplement: Supplementary file 1 [file antibiotics-15-00345-s001.zip › antibiotics-4179936-supplementary/Corrected supplementary files/Supplementary Table S1.docx]

Supplementary materials for the manuscript entitled:

**Drug-resistant Gram-positive cocci as etiological factors of cardiac implantable electronic device infections – data from the EXTRACT registry**

**Danuta Łoboda^1,2^*,Sylwia Gładysz-Wańha ^2,3^, Michał Joniec^2,3^, Eugeniusz Piłat^2^, Robert D. Wojtyczka^4^, Beata Sarecka-Hujar^5^, Julia Staroń^2^, Denis Swolana^4^, Michał Gibiński^1,2^, Karolina Simionescu^1,2^, Sławomir Wilczyński^5^, and Krzysztof S. Gołba^1,2^**

^1^ Department of Electrocardiology and Heart Failure, Medical University of Silesia in Katowice, 40-635 Katowice, Poland; [dloboda@sum.edu.pl](mailto:dloboda@sum.edu.pl) (D.L.); [mgibinski@sum.edu.pl](mailto:mgibinski@sum.edu.pl) (M.G.); [ksimionescu@sum.edu.pl](mailto:ksimionescu@sum.edu.pl) (K.S.); [kgolba@sum.edu.pl](mailto:kgolba@sum.edu.pl) (K.S.G.). ^2^ Department of Electrocardiology, Upper-Silesian Medical Centre in Katowice, 40-635 Katowice, Poland; [dloboda@sum.edu.pl](mailto:dloboda@sum.edu.pl) (D.L.); [sylwia.gladysz@gmail.com](mailto:sylwia.gladysz@gmail.com) (S.G.-W.); [joniec.michal@gmail.com](mailto:joniec.michal@gmail.com) (M.J.); [eugeniuszpilat@gmail.com](mailto:eugeniuszpilat@gmail.com) (E.P.); [julia.staronelektro@gmail.com](mailto:julia.staronelektro@gmail.com) (J.S.); [mgibinski@sum.edu.pl](mailto:mgibinski@sum.edu.pl) (M.G.); [ksimionescu@sum.edu.pl](mailto:ksimionescu@sum.edu.pl) (K.S.); [kgolba@sum.edu.pl](mailto:kgolba@sum.edu.pl) (K.S.G.). ^3^ Doctoral School of the Medical University of Silesia in Katowice, 40-055 Katowice, Poland; [sylwia.gladysz@gmail.com](mailto:sylwia.gladysz@gmail.com) (S.G.-W.); [joniec.michal@gmail.com](mailto:joniec.michal@gmail.com) (M.J.).

^4^ Department of Microbiology, Faculty of Pharmaceutical Sciences in Sosnowiec, Medical University of Silesia in Katowice, 41-200 Sosnowiec, Poland; [rwojtyczka@sum.edu.pl](mailto:rwojtyczka@sum.edu.pl) (R.D.W.); [dswolana@sum.edu.pl](mailto:dswolana@sum.edu.pl) (D.S.). ^5^ Department of Basic Biomedical Science, Faculty of Pharmaceutical Sciences in Sosnowiec, Medical University of Silesia in Katowice, Poland; [bsarecka-hujar@sum.edu.pl](mailto:bsarecka-hujar@sum.edu.pl) (B.S.-H.); [swilczynski@sum.edu.pl](mailto:swilczynski@sum.edu.pl) (S.W.). ***** Correspondence: [dana.loboda@gmail.com](mailto:dana.loboda@gmail.com) / [dloboda@sum.edu.pl](mailto:dloboda@sum.edu.pl)

**Supplementary** **Table S1**. Definitions of cardiac implantable electronic device-related infection types [18].

| **Infection type** | **Definition** |
| --- | --- |
| Isolated pocket erosion | Skin breakdown with exposure of a fragment of the generator or lead, with or without signs of local inflammation. |
| Isolated pocket infection | Device pocket infection in a patient without systemic signs of infection (including fever, positive blood microbiology results, and vegetations on the CIED leads). |
| Bacteremia | Positive blood cultures with or without systemic infection symptoms and signs. |
| Pocket site infection with bacteremia | Device pocket infection in a patient with positive blood cultures who does not meet the modified Duke's criteria for right-sided endocarditis. |
| Pocket site infection with lead or valvular endocarditis | Systemic infection meeting the modified Duke's criteria for definite or possible right-sided endocarditis, in a patient with concomitant inflammation of the skin and subcutaneous tissue within the CIED pocket/pocket. |
| CIED-related endocarditis without pocket infection | A systemic infection meeting the modified Duke criteria for definite or possible diagnosis of right-sided endocarditis, in a patient with bacteremia from a distant site, without concomitant inflammation of the skin and subcutaneous tissue within the CIED pocket. |

CIED: cardiac implantable electronic device
