# Supplementary material for: Drug-Resistant Gram-Positive Cocci as Etiological Factors of Cardiac Implantable Electronic Device Infections—Data from the EXTRACT Registry
Source: Antibiotics (Basel). 2026 Mar 27;15(4):345. doi: 10.3390/antibiotics15040345 (PMC13113275; doi:10.3390/antibiotics15040345)
Supplement: Supplementary file 1 [file antibiotics-15-00345-s001.zip › antibiotics-4179936-supplementary/Corrected supplementary files/Supplementary Table S1.pdf]

Supplementary materials for the manuscript entitled:

Drug-resistant Gram-positive cocci as etiological factors of cardiac implantable electronic device infections – data from the EXTRACT registry

Danuta Łoboda[1,2*], Sylwia Gładysz-Wańha [2,3], Michał Joniec[2,3], Eugeniusz Piłat[2], Robert D. Wojtyczka[4], Beata Sarecka-Hujar[5], Julia Staroń[2], Denis Swolana[4], Michał Gibiński[1,2], Karolina Simionescu[1,2], Sławomir Wilczyński[5], and Krzysztof S. Gołba[1,2]

[1] Department of Electrocardiology and Heart Failure, Medical University of Silesia in Katowice, 40-635 Katowice, Poland; dloboda@sum.edu.pl (D.L.); mgibinski@sum.edu.pl (M.G.); ksimionescu@sum.edu.pl (K.S.); kgolba@sum.edu.pl (K.S.G.). [2] Department of Electrocardiology, Upper-Silesian Medical Centre in Katowice, 40-635 Katowice, Poland; dloboda@sum.edu.pl (D.L.); sylwia.gladysz@gmail.com (S.G.-W.); joniec.michal@gmail.com (M.J.); eugeniuszpilat@gmail.com (E.P.); julia.staronelektro@gmail.com (J.S.); mgibinski@sum.edu.pl (M.G.); ksimionescu@sum.edu.pl (K.S.); kgolba@sum.edu.pl (K.S.G.). [3] Doctoral School of the Medical University of Silesia in Katowice, 40-055 Katowice, Poland; sylwia.gladysz@gmail.com (S.G.-W.); joniec.michal@gmail.com (M.J.). [4] Department of Microbiology, Faculty of Pharmaceutical Sciences in Sosnowiec, Medical University of Silesia in Katowice, 41-200 Sosnowiec, Poland; rwojtyczka@sum.edu.pl (R.D.W.); dswolana@sum.edu.pl (D.S.). [5] Department of Basic Biomedical Science, Faculty of Pharmaceutical Sciences in Sosnowiec, Medical University of Silesia in Katowice, Poland; bsarecka-hujar@sum.edu.pl (B.S.-H.); swilczynski@sum.edu.pl (S.W.). * Correspondence: dana.loboda@gmail.com / dloboda@sum.edu.pl

Supplementary Table S1. Definitions of cardiac implantable electronic device-related infection types [18].

Infection type	Definition
Isolated pocket erosion	Skin breakdown with exposure of a fragment of the generator or lead, with or without signs of local inflammation.
Isolated pocket infection	Device pocket infection in a patient without systemic signs of infection (including fever, positive blood microbiology results, and vegetations on the CIED leads).
Bacteremia	Positive blood cultures with or without systemic infection symptoms and signs.
Pocket site infection with bacteremia	Device pocket infection in a patient with positive blood cultures who does not meet the modified Duke's criteria for right-sided endocarditis.
Pocket site infection with lead or valvular endocarditis	Systemic infection meeting the modified Duke's criteria for definite or possible right-sided endocarditis, in a patient with concomitant inflammation of the skin and subcutaneous tissue within the CIED pocket/pocket.
CIED-related endocarditis without pocket infection	A systemic infection meeting the modified Duke criteria for definite or possible diagnosis of right-sided endocarditis, in a patient with bacteremia from a distant site, without concomitant inflammation of the skin and subcutaneous tissue within the CIED pocket.

CIED: cardiac implantable electronic device
