# Supplementary material for: Drug-Resistant Gram-Positive Cocci as Etiological Factors of Cardiac Implantable Electronic Device Infections—Data from the EXTRACT Registry
Source: Antibiotics (Basel). 2026 Mar 27;15(4):345. doi: 10.3390/antibiotics15040345 (PMC13113275; doi:10.3390/antibiotics15040345)
Supplement: Supplementary file 1 [file antibiotics-15-00345-s001.zip › antibiotics-4179936-supplementary/Corrected supplementary files/Supplementary Table S2.pdf]

Supplementary materials for the manuscript entitled:

Drug-resistant Gram-positive cocci as etiological factors of cardiac implantable electronic device infections – data from the EXTRACT registry

Danuta Łoboda[1,2*],Sylwia Gładysz-Wańha [2,3], Michał Joniec[2,3], Eugeniusz Piłat[2], Robert D. Wojtyczka[4], Beata Sarecka-Hujar[5], Julia Staroń[2], Denis Swolana[4], Michał Gibiński[1,2], Karolina Simionescu[1,2], Sławomir Wilczyński[5], and Krzysztof S. Gołba[1,2]

[1] Department of Electrocardiology and Heart Failure, Medical University of Silesia in Katowice, 40-635 Katowice, Poland; dloboda@sum.edu.pl (D.L.); mgibinski@sum.edu.pl (M.G.); ksimionescu@sum.edu.pl (K.S.); kgolba@sum.edu.pl (K.S.G.). [2] Department of Electrocardiology, Upper-Silesian Medical Centre in Katowice, 40-635 Katowice, Poland; dloboda@sum.edu.pl (D.L.); sylwia.gladysz@gmail.com (S.G.-W.); joniec.michal@gmail.com (M.J.); eugeniuszpilat@gmail.com (E.P.); julia.staronelektro@gmail.com (J.S.); mgibinski@sum.edu.pl (M.G.); ksimionescu@sum.edu.pl (K.S.); kgolba@sum.edu.pl (K.S.G.). [3] Doctoral School of the Medical University of Silesia in Katowice, 40-055 Katowice, Poland; sylwia.gladysz@gmail.com (S.G.-W.); joniec.michal@gmail.com (M.J.). [4] Department of Microbiology, Faculty of Pharmaceutical Sciences in Sosnowiec, Medical University of Silesia in Katowice, 41-200 Sosnowiec, Poland; rwojtyczka@sum.edu.pl (R.D.W.); dswolana@sum.edu.pl (D.S.). [5] Department of Basic Biomedical Science, Faculty of Pharmaceutical Sciences in Sosnowiec, Medical University of Silesia in Katowice, Poland; bsarecka-hujar@sum.edu.pl (B.S.-H.); swilczynski@sum.edu.pl (S.W.). * Correspondence: dana.loboda@gmail.com / dloboda@sum.edu.pl

Supplementary Table S2. Resistance patterns in methicillin-susceptible and methicillin-resistant staphylococci.

		E	DA	GM	CIP	LEV	TE	TGC	VA	SXT	RIF	LNZ	DAP	**MDR**
MS *S. aureus*, n (%)	S	48 (82.8)	50 (86.2)	53 (91.4)	27 (46.6)	27 (46.6)	49 (90.7)	58 (100.0)	58 (100.0)	57 (98.3)	58 (100.0)	58 (100.0)	58 (100.0)	**1 (1.7)**
	I	0 (0.0)	0 (0.0)	0 (0.0)	28 (48.3)	28 (48.3)	1 (1.9)	0 (0.0)	0 (0.0)	0 (0.0)	0 (0.0)	0 (0.0)	0 (0.0)	
	R	10 (17.2)	8 (13.8)	5 (8.6)	3 (5.2)	3 (5.2)	4 (7.4)	0 (0.0)	0 (0.0)	1 (1.7)	0 (0.0)	0 (0.0)	0 (0.0)	
MS CoNS, n (%)	S	52 (67.5)	59 (75.6)	71 (91.0)	40 (51.3)	40 (51.3)	54 (69.2)	77 (98.7)	78 (100.0)	73 (93.6)	77 (100.0)	78 (100.0)	75 (98.7)	**8 (10.4)**
	I	0 (0.0)	1 (1.3)	0 (0.0)	34 (43.6)	34 (43.6)	15 (19.2)	0 (0.0)	0 (0.0)	5 (6.4)	0 (0.0)	0 (0.0)	0 (0.0)	
	R	25 (32.5)	18 (23.1)	7 (9.0)	4 (5.1)	4 (5.1)	9 (11.5)	1 (1.3)	0 (0.0)	0 (0.0)	0 (0.0)	0 (0.0)	1 (1.3)	

		E	DA	GM	CIP	LEV	TE	TGC	VA	SXT	RIF	LNZ	DAP	MDR
MR *S. aureus*, n (%)	S	0 (0.0)	0 (0.0)	4 (100.0)	0 (0.0)	0 (0.0)	4 (100.0)	4 (100.0)	4 (100.0)	4 (100.0)	4 (100.0)	3 (75.0)	4 (100.0)	**4 (100.0)**
	I	0 (0.0)	0 (0.0)	0 (0.0)	0 (0.0)	0 (0.0)	0 (0.0)	0 (0.0)	0 (0.0)	0 (0.0)	0 (0.0)	0 (0.0)	0 (0.0)	
	R	4 (100.0)	4 (100.0)	0 (0.0)	4 (100.0)	4 (100.0)	0 (0.0)	0 (0.0)	0 (0.0)	0 (0.0)	0 (0.0)	1 (25.0)	0 (0.0)	
MR CoNS, n (%)	S	26 (26.8)	46 (46.9)	58 (58.6)	23 (23.5)	24 (24.7)	31 (31.6)	96 (99.0)	99 (100.0)	57 (57.6)	85 (90.4)	97 (98.0)	89 (94.7)	**74 (75.5)**
	I	0 (0.0)	2 (2.0)	0 (0.0)	20 (20.4)	22 (22.7)	38 (38.8)	0 (0.0)	0 (0.0)	13 (13.1)	0 (0.0)	0 (0.0)	0 (0.0)	
	R	71 (73.2)	50 (51.0)	41 (41.4)	55 (56.1)	51 (52.6)	29 (29.6)	1 (1.0)	0 (0.0)	29 (29.3)	9 (9.6)	2 (2.0)	5 (5.3)	

CoNS: coagulase negative staphylococci; I: susceptible, increased exposure; MDR: multidrug-resistant; MR: methicillin-resistant; MS: methicillin-susceptible; R: resistant; S: susceptible. standard dosing regimen. The abbreviations for antibiotics are from the European Committee on Antimicrobial Susceptibility Testing Breakpoint tables for interpretation of Minimum Inhibitory Concentrations and zone diameters Version 15.0. valid from 2025-01-01 [17]: CIP: ciprofloxacin; DA: clindamycin; DAP: daptomycin; E: erythromycin; GM: gentamicin; LEV: levofloxacin; LNZ: linezolid; QDA: quinupristin/dalfopristin; RIF: rifampicin; SPT: spectinomycin; SXT: trimethoprim-sulfamethoxazole; TE: tetracycline; TEC: teicoplanin; TGC: tigecycline; VA: vancomycin
