# Supplementary material for: Drug-Resistant Gram-Positive Cocci as Etiological Factors of Cardiac Implantable Electronic Device Infections—Data from the EXTRACT Registry
Source: Antibiotics (Basel). 2026 Mar 27;15(4):345. doi: 10.3390/antibiotics15040345 (PMC13113275; doi:10.3390/antibiotics15040345)
Supplement: Supplementary file 1 [file antibiotics-15-00345-s001.zip › antibiotics-4179936-supplementary/Corrected supplementary files/Supplementary Table S4.docx]

Supplementary materials for the manuscript entitled:

**Drug-resistant Gram-positive cocci as etiological factors of cardiac implantable electronic device infections – data from the EXTRACT registry**

**Danuta Łoboda^1,2^*,Sylwia Gładysz-Wańha ^2,3^, Michał Joniec^2,3^, Eugeniusz Piłat^2^, Robert D. Wojtyczka^4^, Beata Sarecka-Hujar^5^, Julia Staroń^2^, Denis Swolana^4^, Michał Gibiński^1,2^, Karolina Simionescu^1,2^, Sławomir Wilczyński^5^, and Krzysztof S. Gołba^1,2^**

^1^ Department of Electrocardiology and Heart Failure, Medical University of Silesia in Katowice, 40-635 Katowice, Poland; [dloboda@sum.edu.pl](mailto:dloboda@sum.edu.pl) (D.L.); [mgibinski@sum.edu.pl](mailto:mgibinski@sum.edu.pl) (M.G.); [ksimionescu@sum.edu.pl](mailto:ksimionescu@sum.edu.pl) (K.S.); [kgolba@sum.edu.pl](mailto:kgolba@sum.edu.pl) (K.S.G.). ^2^ Department of Electrocardiology, Upper-Silesian Medical Centre in Katowice, 40-635 Katowice, Poland; [dloboda@sum.edu.pl](mailto:dloboda@sum.edu.pl) (D.L.); [sylwia.gladysz@gmail.com](mailto:sylwia.gladysz@gmail.com) (S.G.-W.); [joniec.michal@gmail.com](mailto:joniec.michal@gmail.com) (M.J.); [eugeniuszpilat@gmail.com](mailto:eugeniuszpilat@gmail.com) (E.P.); [julia.staronelektro@gmail.com](mailto:julia.staronelektro@gmail.com) (J.S.); [mgibinski@sum.edu.pl](mailto:mgibinski@sum.edu.pl) (M.G.); [ksimionescu@sum.edu.pl](mailto:ksimionescu@sum.edu.pl) (K.S.); [kgolba@sum.edu.pl](mailto:kgolba@sum.edu.pl) (K.S.G.). ^3^ Doctoral School of the Medical University of Silesia in Katowice, 40-055 Katowice, Poland; [sylwia.gladysz@gmail.com](mailto:sylwia.gladysz@gmail.com) (S.G.-W.); [joniec.michal@gmail.com](mailto:joniec.michal@gmail.com) (M.J.). ^4^ Department of Microbiology, Faculty of Pharmaceutical Sciences in Sosnowiec, Medical University of Silesia in Katowice, 41-200 Sosnowiec, Poland; [rwojtyczka@sum.edu.pl](mailto:rwojtyczka@sum.edu.pl) (R.D.W.); [dswolana@sum.edu.pl](mailto:dswolana@sum.edu.pl) (D.S.).
^5^ Department of Basic Biomedical Science, Faculty of Pharmaceutical Sciences in Sosnowiec, Medical University of Silesia in Katowice, Poland; [bsarecka-hujar@sum.edu.pl](mailto:bsarecka-hujar@sum.edu.pl) (B.S.-H.); [swilczynski@sum.edu.pl](mailto:swilczynski@sum.edu.pl) (S.W.). ***** Correspondence: [dana.loboda@gmail.com](mailto:dana.loboda@gmail.com) / [dloboda@sum.edu.pl](mailto:dloboda@sum.edu.pl)

**Supplementary Table S4.**

**The RECORD statement – checklist of items, extended from the STROBE statement, that should be reported in observational studies using routinely collected health data.**

|  | **Item No.** | **STROBE items** | **Location in manuscript where items are reported** | **RECORD items** | **Location in manuscript where items are reported** |
| --- | --- | --- | --- | --- | --- |
| **Title and abstract** | | | | | |
|  | 1 | (a) Indicate the study’s design with a commonly used term in the title or the abstract (b) Provide in the abstract an informative and balanced summary of what was done and what was found | Pg 1 Title  Pg 1 Abstract | RECORD 1.1: The type of data used should be specified in the title or abstract. When possible, the name of the databases used should be included.  RECORD 1.2: If applicable, the geographic region and timeframe within which the study took place should be reported in the title or abstract.  RECORD 1.3: If linkage between databases was conducted for the study, this should be clearly stated in the title or abstract. | Pg 1 Title  Pg 1 Abstract  Pg 12  4. Materials and Methods,  4.1. Study Group  Not applicable |
| **Introduction** | | | | | |
| Background rationale | 2 | Explain the scientific background and rationale for the investigation being reported | Pg 2  1. Introduction |  |  |
| Objectives | 3 | State specific objectives, including any prespecified hypotheses | Pg 2  1. Introduction |  |  |
| **Methods** | | | | | |
| Study Design | 4 | Present key elements of study design early in the paper | Pg 12-13  4. Materials and Methods,  4.1. Study Group, 4.3. Operating and microbiological procedures |  |  |
| Setting | 5 | Describe the setting, locations, and relevant dates, including periods of recruitment, exposure, follow-up, and data collection | Pg 12-13  4. Materials and Methods,  4.1. Study Group  Follow‑up: not applicable (registry-based, single-episode infection data) |  |  |
| Participants | 6 | **(a) Cohort study -** Give the eligibility criteria, and the sources and methods of selection of participants. Describe methods of follow-up  *Case-control study* - Give the eligibility criteria, and the sources and methods of case ascertainment and control selection. Give the rationale for the choice of cases and controls  *Cross-sectional study* - Give the eligibility criteria, and the sources and methods of selection of participants  *(b) Cohort study* - For matched studies, give matching criteria and number of exposed and unexposed  *Case-control study* - For matched studies, give matching criteria and the number of controls per case | **(a) Cohort study**  Pg 12-13  4. Materials and Methods,  4.1. Study Group  Follow‑up: not applicable (registry-based, single-episode infection data)  Not applicable | RECORD 6.1: The methods of study population selection (such as codes or algorithms used to identify subjects) should be listed in detail. If this is not possible, an explanation should be provided.  RECORD 6.2: Any validation studies of the codes or algorithms used to select the population should be referenced. If validation was conducted for this study and not published elsewhere, detailed methods and results should be provided.  RECORD 6.3: If the study involved linkage of databases, consider use of a flow diagram or other graphical display to demonstrate the data linkage process, including the number of individuals with linked data at each stage. | Pg 12  4. Materials and Methods,  4.1. Study Group  Pg 13  4. Materials and Methods,  4.2. Data extraction  Not applicable |
| Variables | 7 | Clearly define all outcomes, exposures, predictors, potential confounders, and effect modifiers. Give diagnostic criteria, if applicable. | Pg 12  3. Discussion,  3.7. Study limitations  Supplementary Diagnostic criteria: Table S1. Definitions of cardiac implantable electronic device-related infection types | RECORD 7.1: A complete list of codes and algorithms used to classify exposures, outcomes, confounders, and effect modifiers should be provided. If these cannot be reported, an explanation should be provided. | Outcomes: MDR occurrence in isolates, resistance profile. Tables 3-5, Supplementary Tables S2-S3,  Figures 1-3. Exposures: not analyzed.  Predictors: type of infection, prevalence of MDR strains in a given year. Table 2, Supplementary Figures S1-S4.  Potential confounders: age, gender, BMI, comorbidities, sample contamination. Table 1.  Effect modifiers: pathogen species. Tables 3-5, Supplementary Tables S2-S3, Suppl. Figures S1-S4. |
| Data sources/ measurement | 8 | For each variable of interest, give sources of data and details of methods of assessment (measurement).  Describe comparability of assessment methods if there is more than one group | Source of all data: EXTRACT registry  Pg 12  4. Materials and Methods,  4.1. Study Group  One group |  |  |
| Bias | 9 | Describe any efforts to address potential sources of bias | Pg 12-13  4. Materials and Methods,  4.1. Study Group  4.2. Data extraction and quality assessment |  |  |
| Study size | 10 | Explain how the study size was arrived at | No formal sample size calculation was performed. The study size was determined by the number of consecutive cases recorded in the EXTRACT registry during the study period (from 2016 to June 2025) and the prevalence of CIED infections in this timeframe. |  |  |
| Quantitative variables | 11 | Explain how quantitative variables were handled in the analyses. If applicable, describe which groupings were chosen, and why | Pg 13  4. Materials and Methods,  4.4. Statistical analysis |  |  |
| Statistical methods | 12 | (a) Describe all statistical methods, including those used to control for confounding  (b) Describe any methods used to examine subgroups and interactions  (c) Explain how missing data were addressed  (d) *Cohort study* - If applicable, explain how loss to follow-up was addressed  *Case-control study* - If applicable, explain how matching of cases and controls was addressed  *Cross-sectional study* - If applicable, describe analytical methods taking account of sampling strategy  (e) Describe any sensitivity analyses | Pg 13  4. Materials and Methods,  4.4. Statistical analysis |  |  |
| Data access and cleaning methods |  | .. |  | RECORD 12.1: Authors should describe the extent to which the investigators had access to the database population used to create the study population.  RECORD 12.2: Authors should provide information on the data cleaning methods used in the study. | Researchers had full access to the population database used to create the study population.  4. Materials and Methods,  4.2. Data extraction and quality assessment |
| Linkage |  | .. |  | RECORD 12.3: State whether the study included person-level, institutional-level, or other data linkage across two or more databases. The methods of linkage and methods of linkage quality evaluation should be provided. | Not applicable |
| **Results** | | | | | |
| Participants | 13 | (a) Report the numbers of individuals at each stage of the study (*e.g.*, numbers potentially eligible, examined for eligibility, confirmed eligible, included in the study, completing follow-up, and analysed)  (b) Give reasons for non-participation at each stage.  (c) Consider use of a flow diagram | Pg 12  4. Materials and Methods,  4.1. Study Group | RECORD 13.1: Describe in detail the selection of the persons included in the study (*i.e.,* study population selection) including filtering based on data quality, data availability and linkage. The selection of included persons can be described in the text and/or by means of the study flow diagram. | Pg 12  4. Materials and Methods,  4.1. Study Group |
| Descriptive data | 14 | (a) Give characteristics of study participants (*e.g.*, demographic, clinical, social) and information on exposures and potential confounders  (b) Indicate the number of participants with missing data for each variable of interest  (c) *Cohort study* - summarise follow-up time (*e.g.*, average and total amount) | (a) Table 1.  (b) Cases with negative cultures were not excluded from the cohort. This data is important from an epidemiological perspective.  (c) Not applicable |  |  |
| Outcome data | 15 | *Cohort study* - Report numbers of outcome events or summary measures over time  *Case-control study* - Report numbers in each exposure category, or summary measures of exposure  *Cross-sectional study* - Report numbers of outcome events or summary measures | 2. Results,  2.4. MDR strains  Tables 3-5, Supplementary Tables S2-S3.  Figures 1-3,  Suppl. Figures S1-S4. |  |  |
| Main results | 16 | (a) Give unadjusted estimates and, if applicable, confounder-adjusted estimates and their precision (e.g., 95% confidence interval). Make clear which confounders were adjusted for and why they were included  (b) Report category boundaries when continuous variables were categorized  (c) If relevant, consider translating estimates of relative risk into absolute risk for a meaningful time period | Table 2.  Not applicable  Not applicable |  |  |
| Other analyses | 17 | Report other analyses done—e.g., analyses of subgroups and interactions, and sensitivity analyses | Not applicable |  |  |
| **Discussion** | | | | | |
| Key results | 18 | Summarise key results with reference to study objectives | Pg 7  3. Discussion |  |  |
| Limitations | 19 | Discuss limitations of the study, taking into account sources of potential bias or imprecision. Discuss both direction and magnitude of any potential bias | Pg 12  3. Discussion,  3.7. Study limitations | RECORD 19.1: Discuss the implications of using data that were not created or collected to answer the specific research question(s). Include discussion of misclassification bias, unmeasured confounding, missing data, and changing eligibility over time, as they pertain to the study being reported. | Pg 12  3. Discussion,  3.7. Study limitations |
| Interpretation | 20 | Give a cautious overall interpretation of results considering objectives, limitations, multiplicity of analyses, results from similar studies, and other relevant evidence | Pg 7-12  3. Discussion |  |  |
| Generalisability | 21 | Discuss the generalisability (external validity) of the study results | Pg 12  3. Discussion,  3.7. Study limitations |  |  |
| **Other Information** | | | | | |
| Funding | 22 | Give the source of funding and the role of the funders for the present study and, if applicable, for the original study on which the present article is based | Pg 14  Funding |  |  |
| Accessibility of protocol, raw data, and programming code |  | .. | Pg 14  Data Availability Statement | RECORD 22.1: Authors should provide information on how to access any supplemental information such as the study protocol, raw data, or programming code. | Pg 14  Data Availability Statement |

*Checklist is protected under Creative Commons Attribution ([CC BY](http://creativecommons.org/licenses/by/4.0/)) license.
