# Supplementary figures and images for: Drug-Resistant Gram-Positive Cocci as Etiological Factors of Cardiac Implantable Electronic Device Infections—Data from the EXTRACT Registry
Source: Antibiotics (Basel). 2026 Mar 27;15(4):345. doi: 10.3390/antibiotics15040345 (PMC13113275; doi:10.3390/antibiotics15040345)

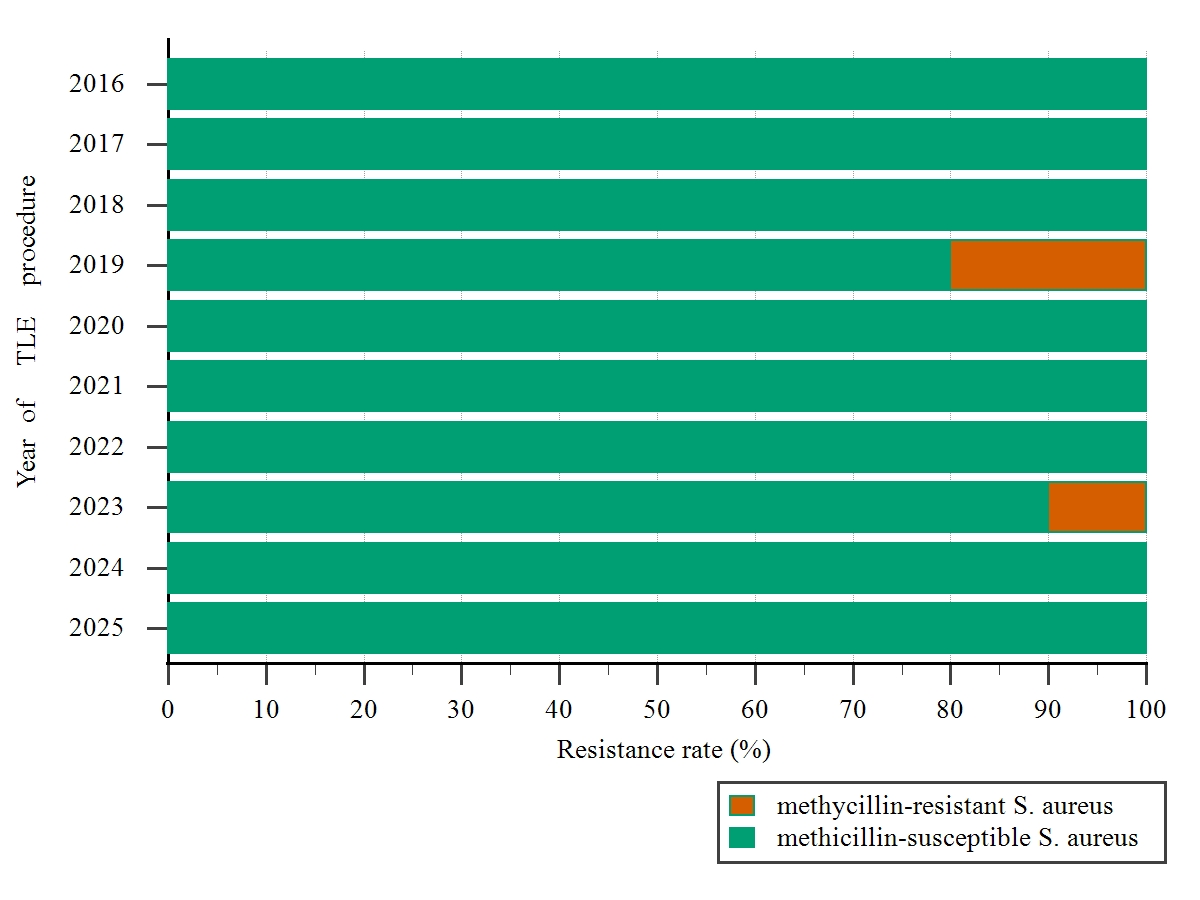

Supplement: Supplementary file 1 [file antibiotics-15-00345-s001.zip › antibiotics-4179936-supplementary/Corrected supplementary files/Suppl. Fig. S1A. Methicillin resistance rate in Staphylococcus aureus from 2016 ro 2025..jpg]

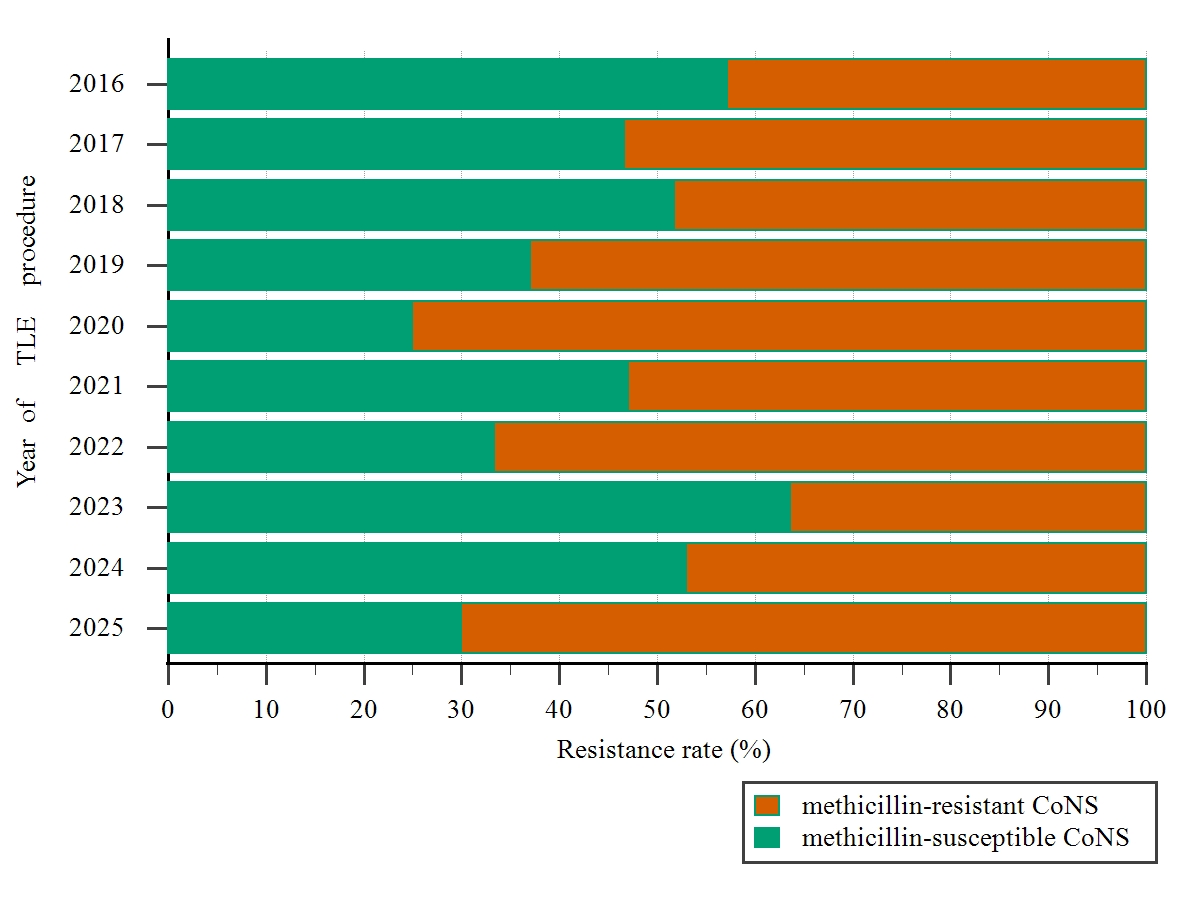

Supplement: Supplementary file 1 [file antibiotics-15-00345-s001.zip › antibiotics-4179936-supplementary/Corrected supplementary files/Suppl. Fig. S1B. Methicillin resistance rate in coagulase-negative staphylococci from 2026 to 2025..jpg]

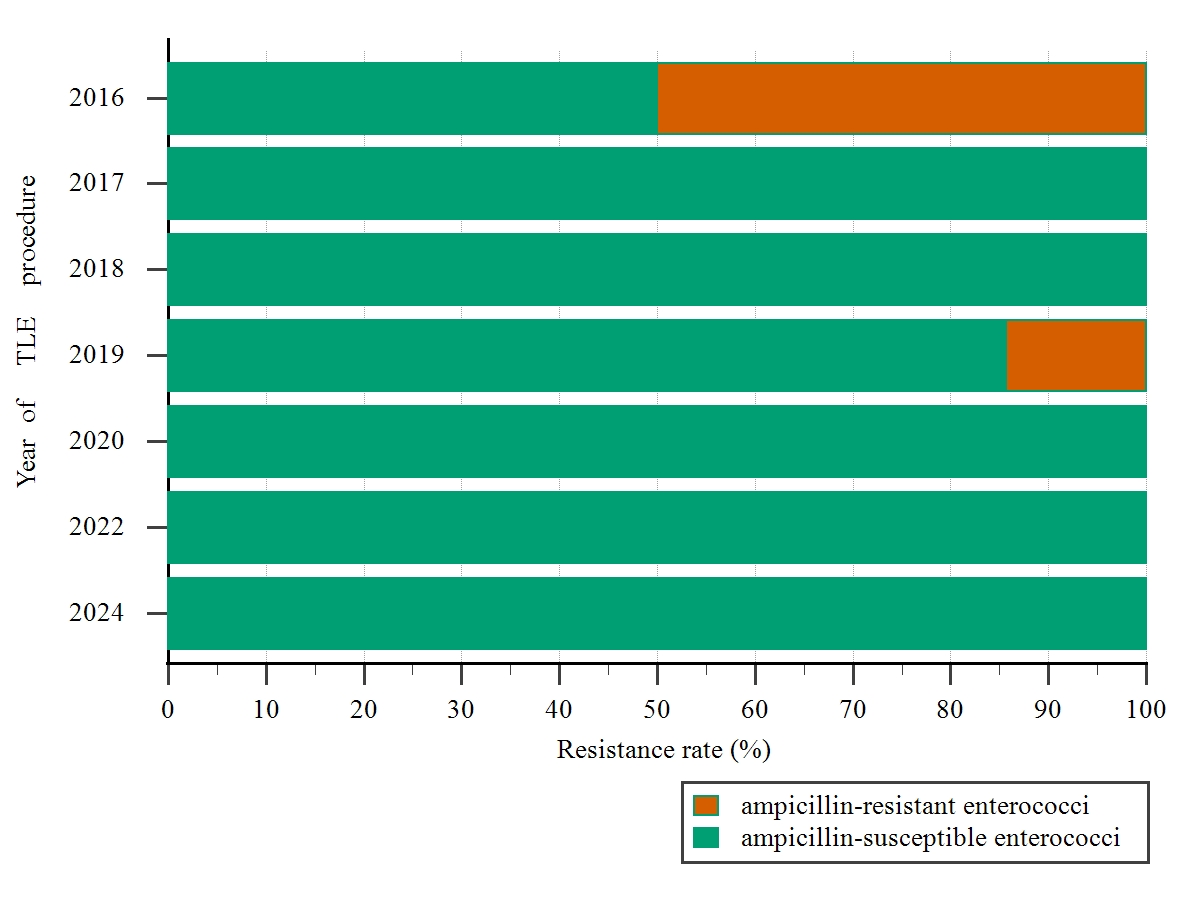

Supplement: Supplementary file 1 [file antibiotics-15-00345-s001.zip › antibiotics-4179936-supplementary/Corrected supplementary files/Suppl. Fig. S1C. Ampicillin resistance rate in enterococci from 2016 to 2025.jpg]

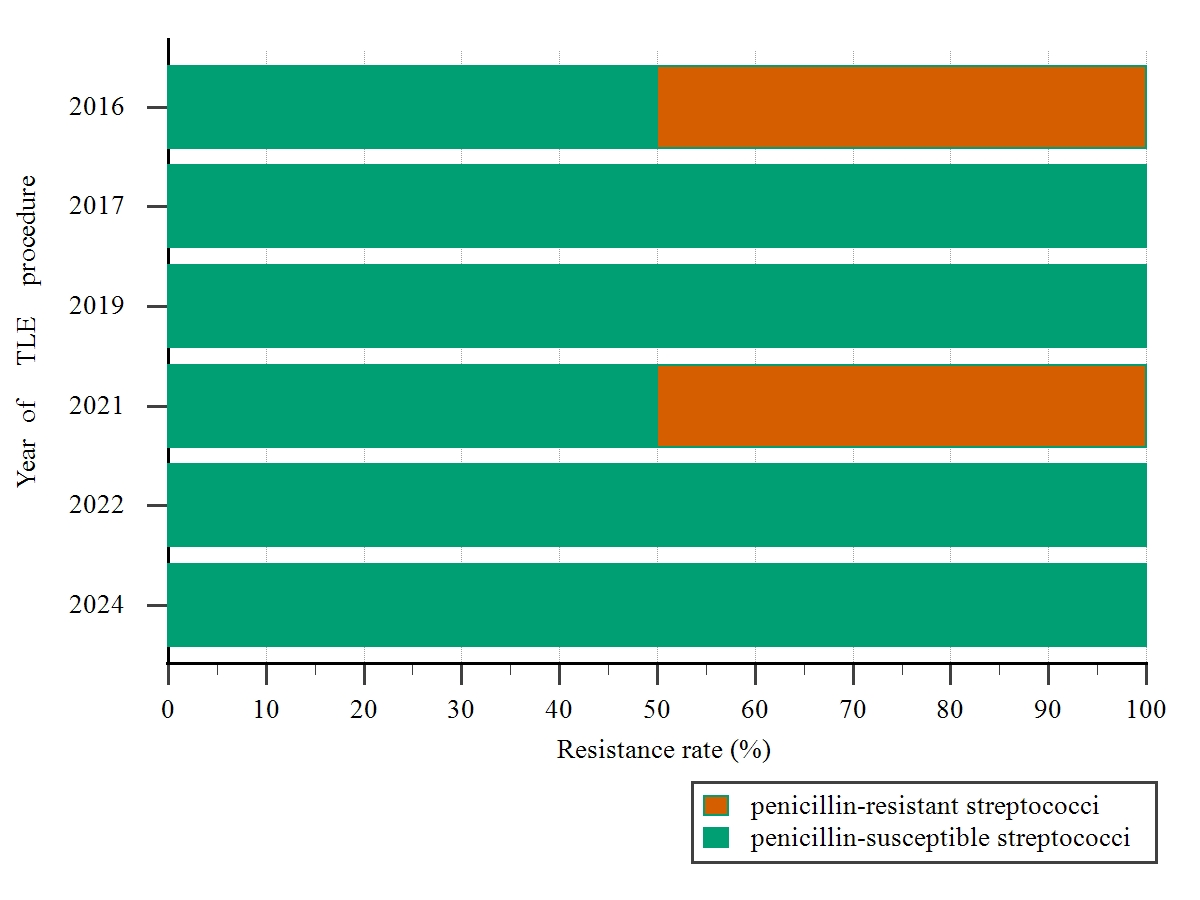

Supplement: Supplementary file 1 [file antibiotics-15-00345-s001.zip › antibiotics-4179936-supplementary/Corrected supplementary files/Suppl. Fig. S1D. Penicillin resistance rate in streptococci from 2016 to 2025.jpg]

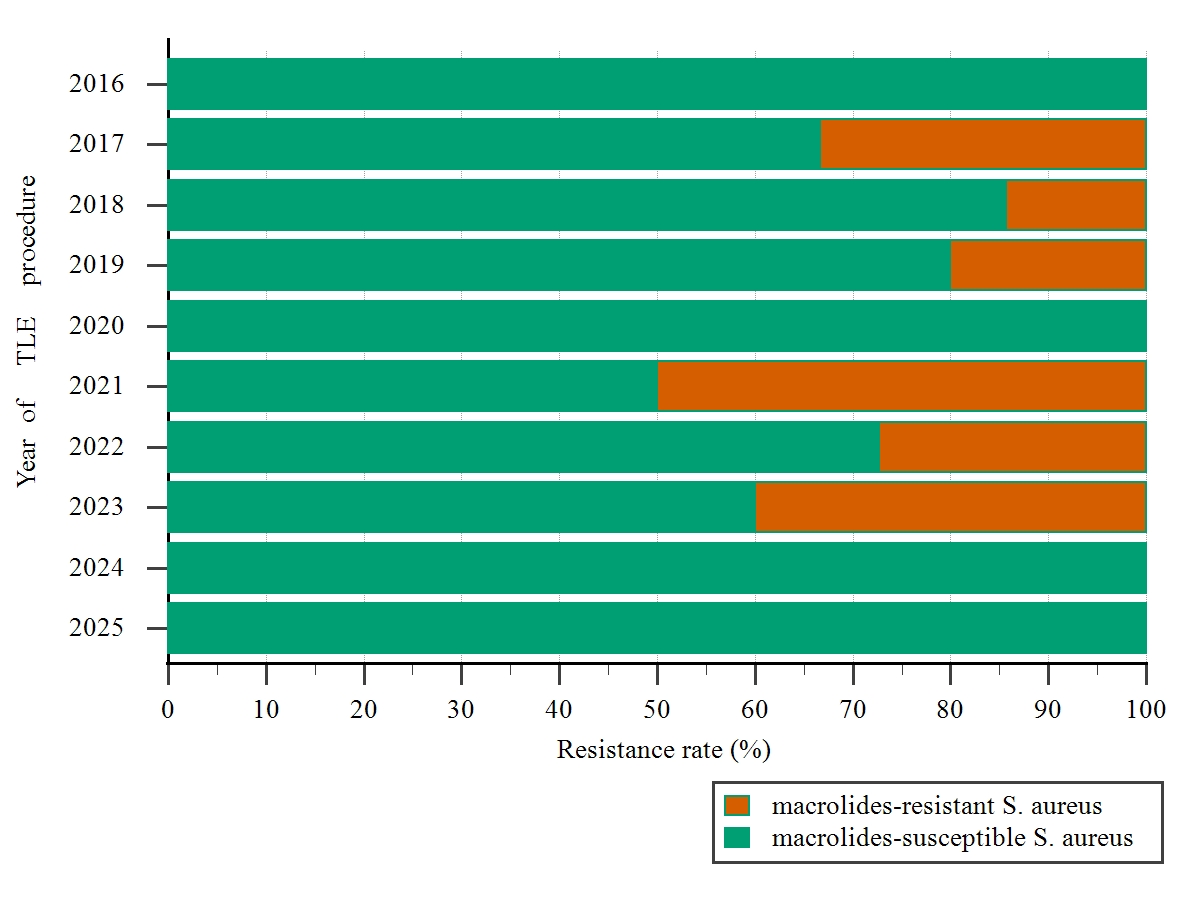

Supplement: Supplementary file 1 [file antibiotics-15-00345-s001.zip › antibiotics-4179936-supplementary/Corrected supplementary files/Suppl. Fig. S2A. Macrolides resistance rate in Staphylococcus aureus from 2016 to 2025.jpg]

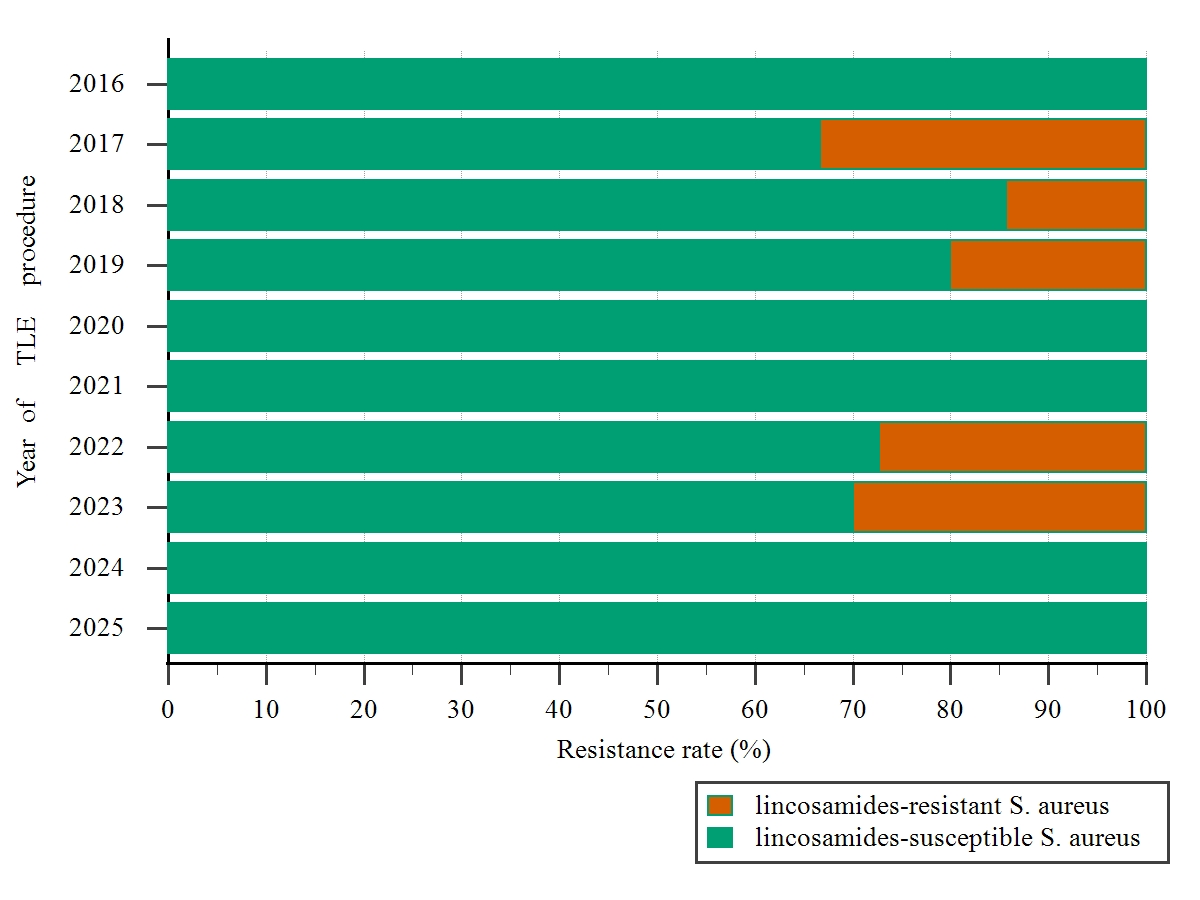

Supplement: Supplementary file 1 [file antibiotics-15-00345-s001.zip › antibiotics-4179936-supplementary/Corrected supplementary files/Suppl. Fig. S2B. Lincosamides resistance rate in Staphylococcus aureus from 2016 to 2025.jpg]

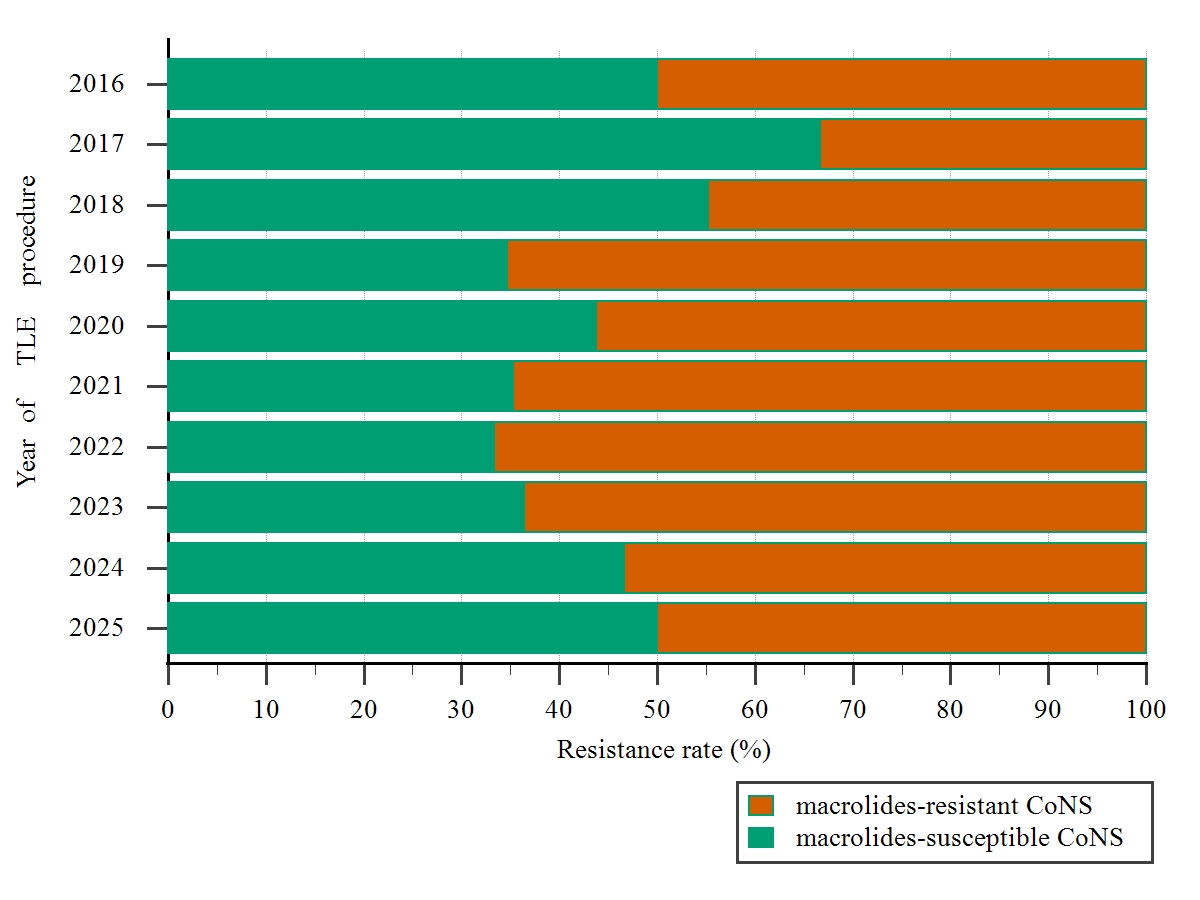

Supplement: Supplementary file 1 [file antibiotics-15-00345-s001.zip › antibiotics-4179936-supplementary/Corrected supplementary files/Suppl. Fig. S2C. Macrolides resistance rate in coagulase-negative staphylococci from 2016 to 2025.jpg]

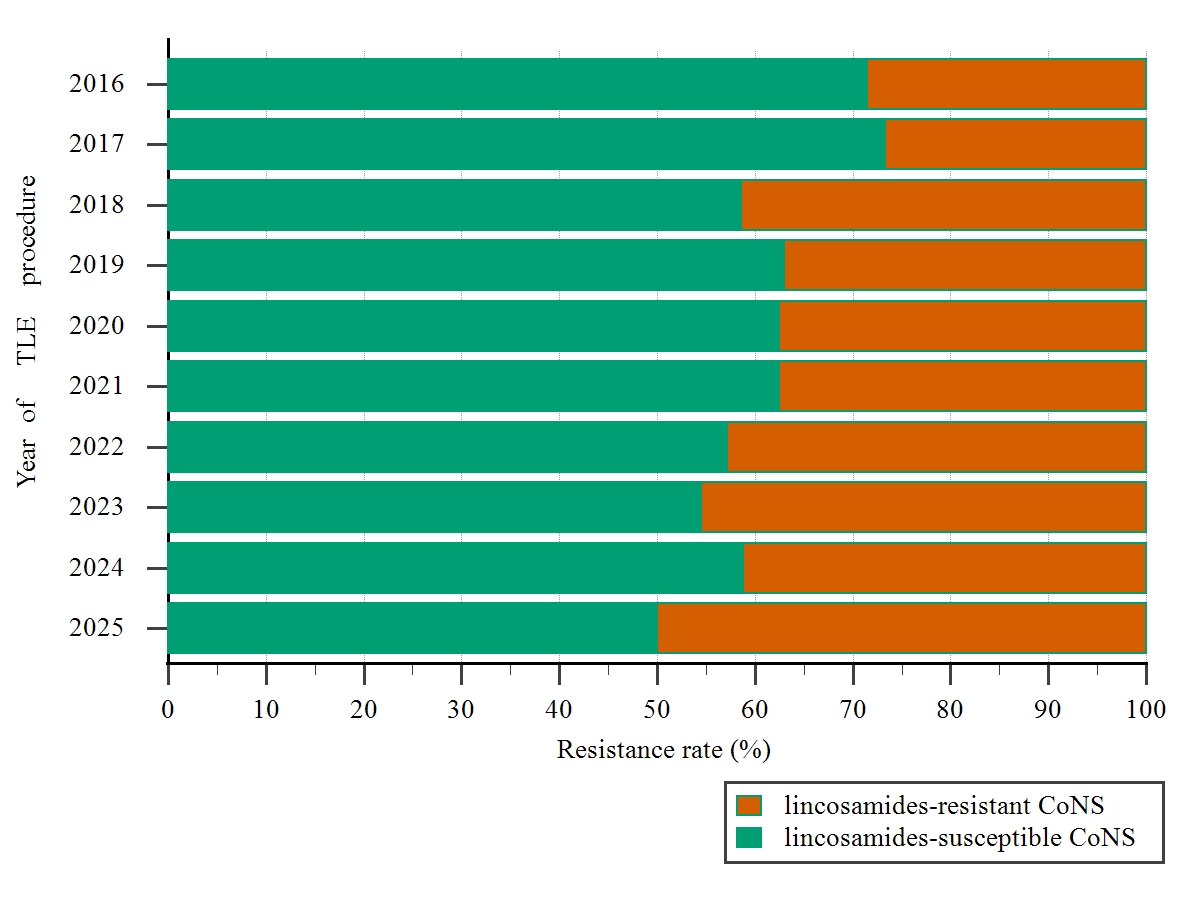

Supplement: Supplementary file 1 [file antibiotics-15-00345-s001.zip › antibiotics-4179936-supplementary/Corrected supplementary files/Suppl. Fig. S2D. Lincosamides resistance rate in coagulase-negative staphylococci from 2016 to 2025.jpg]

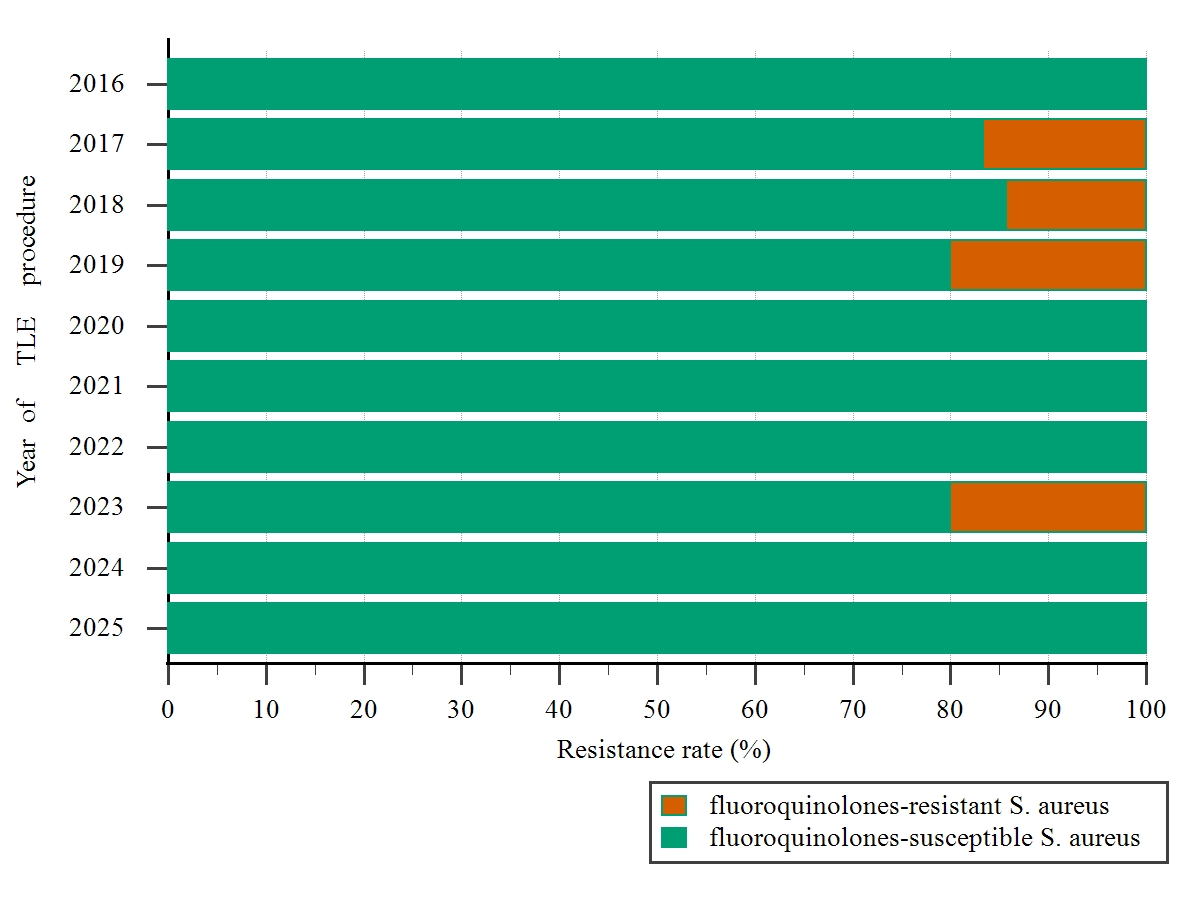

Supplement: Supplementary file 1 [file antibiotics-15-00345-s001.zip › antibiotics-4179936-supplementary/Corrected supplementary files/Suppl. Fig. S3A. Fluoroquinolones resistance rate in Staphylococcus aureus from 2016 to 2025.jpg]

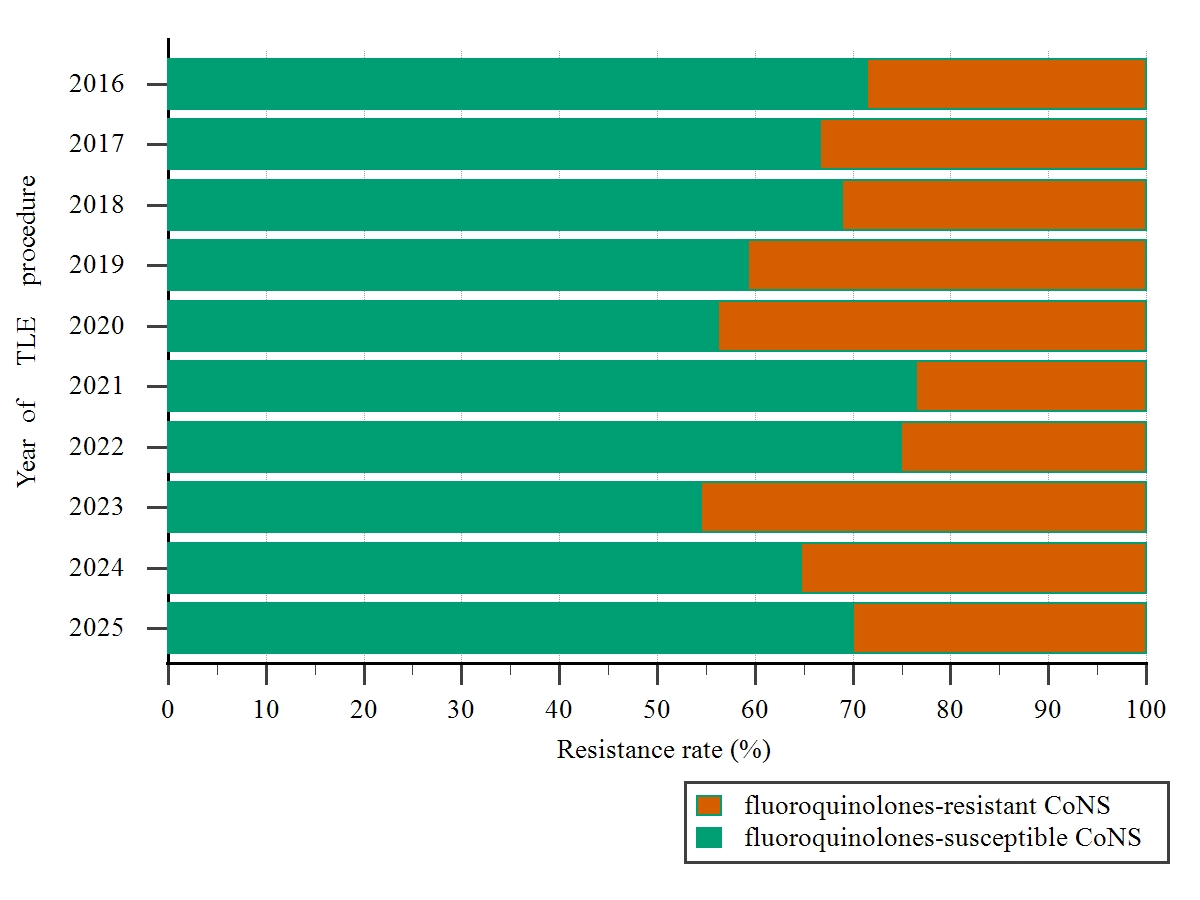

Supplement: Supplementary file 1 [file antibiotics-15-00345-s001.zip › antibiotics-4179936-supplementary/Corrected supplementary files/Suppl. Fig. S3B. Fluoroquinolones resistance rate in coagulase-negative staphylococci from 2016 to 2025.jpg]

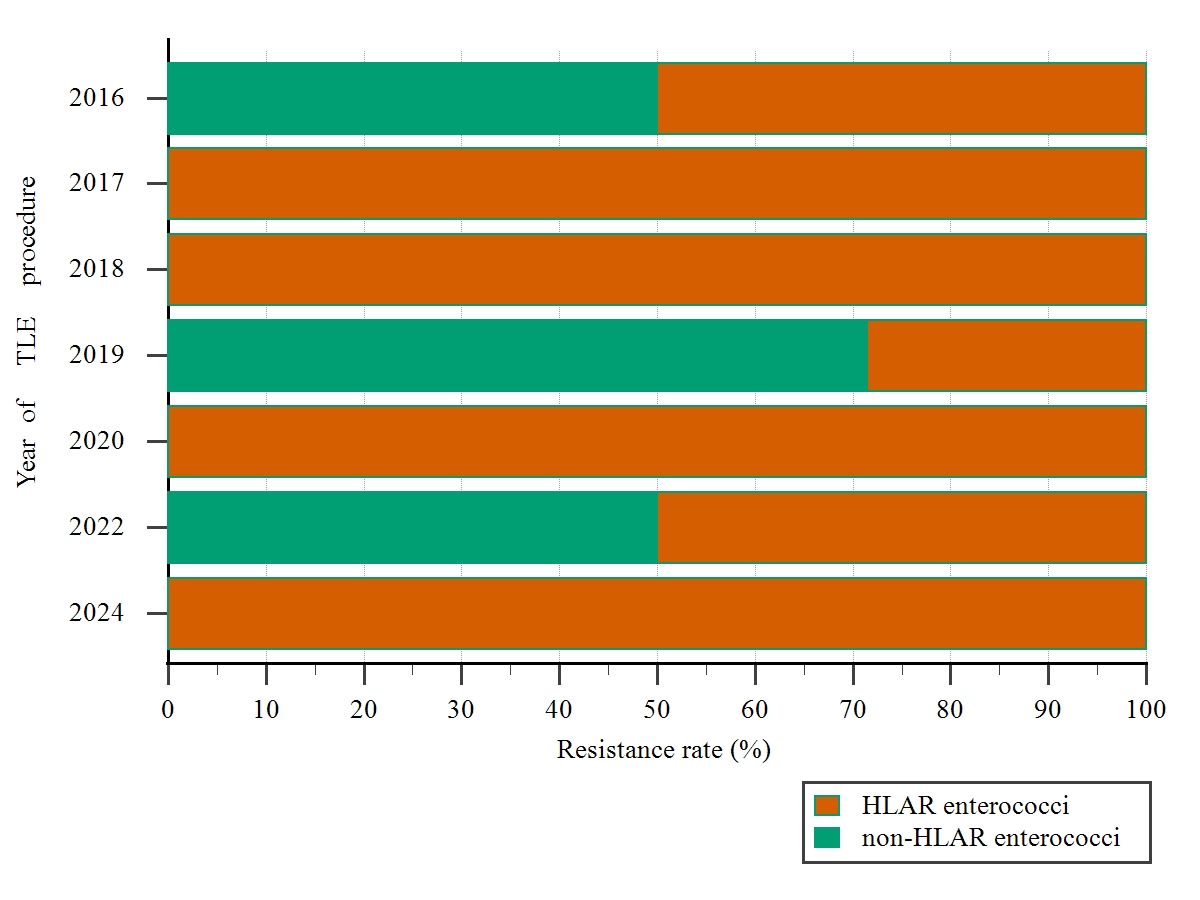

Supplement: Supplementary file 1 [file antibiotics-15-00345-s001.zip › antibiotics-4179936-supplementary/Corrected supplementary files/Suppl. Fig. S4. The high-level aminoglycoside resistance rate in enterococci from 2016 to 2025.jpg]
